# A molecular photosensitizer achieves a *V*_oc_ of 1.24 V enabling highly efficient and stable dye-sensitized solar cells with copper(II/I)-based electrolyte

**DOI:** 10.1038/s41467-021-21945-3

**Published:** 2021-03-19

**Authors:** Dan Zhang, Marko Stojanovic, Yameng Ren, Yiming Cao, Felix T. Eickemeyer, Etienne Socie, Nick Vlachopoulos, Jacques-E. Moser, Shaik M. Zakeeruddin, Anders Hagfeldt, Michael Grätzel

**Affiliations:** 1 Laboratory of Photonics and Interfaces, Institute of Chemical Sciences & Engineering, École Polytechnique Fédérale de Lausanne, Lausanne, Switzerland; 2 Laboratory of Photomolecular Science, Institute of Chemical Sciences & Engineering, École Polytechnique Fédérale de Lausanne, Lausanne, Switzerland; 3 Photochemical Dynamics Group, Institute of Chemical Sciences and Engineering, École Polytechnique Fédérale de Lausanne, Lausanne, Switzerland; 4 Present Address: H.Glass SA, EPFL Innovation Park, Lausanne, Switzerland

**Keywords:** Solar cells, Solar cells

## Abstract

To develop photosensitizers with high open-circuit photovoltage (*V*_oc_) is a crucial strategy to enhance the power conversion efficiency (PCE) of co-sensitized solar cells. Here, we show a judiciously tailored organic photosensitizer, coded MS5, featuring the bulky donor *N*-(2’,4’-bis(dodecyloxy)-[1,1’-biphenyl]-4-yl)-2’,4’-bis(dodecyloxy)-*N*-phenyl-[1,1’-biphenyl]-4-amine and the electron acceptor 4-(benzo[*c*][1,2,5]thiadiazol-4-yl)benzoic acid. Employing MS5 with a copper (II/I) electrolyte enables a dye-sensitized solar cell (DSC) to achieve a strikingly high *V*_oc_ of 1.24 V, with the *V*_oc_ deficit as low as 130 mV and an ideality factor of merely 1.08. The co-sensitization of MS5 with the wider spectral-response dye XY1b produces a highly efficient and stable DSC with the PCE of 13.5% under standard AM1.5 G, 100 mW cm^−2^ solar radiation. Remarkably, the co-sensitized solar cell (active area of 2.8 cm^2^) presents a record PCE of 34.5% under ambient light, rendering it very attractive as an ambient light harvesting energy source for low power electronics.

## Abstract (web summary)

Designing photo-sensitisers with high open-circuit voltage (*V*_oc_) is desirable to enhance the power conversion efficiency (PCE) of co-sensitized solar cells. Here, the authors employ a judiciously tailored organic sensitiser MS5 with copper electrolyte to achieve a *V*_oc_ of 1.24 V, and recorded PCE of 34.5% under ambient light.

## Introduction

Since the industrial revolution, tremendous consumption of fossil fuels has led to rapid growth of the global economy and improved quality of life, but at the cost of high risks and impacts of climate change due to the CO_2_ emission. The Paris agreement, which aims to hold the increase in the global average temperature to well below 2 °C above pre-industrial levels, relies on the development of renewable energy technologies to reduce the consumption of fossil fuels. Photovoltaics (PV) now supply nearly 3% of global electricity reducing CO_2_ emission and attenuating climate change. Thus, Germany’s PV generated 47.5 TWh electric energy in 2019, covering 8.2% of the country’s gross electricity demand in the same year and avoiding about 29 million tons of CO_2_ emission (https://www.ise.fraunhofer.de/en/publications/studies/photovoltaics-report.html).

Compared to the dominant silicon PV, mesoscopic dye-sensitized solar cells (DSCs) have a lower power conversion efficiency (PCE) under standard AM1.5G conditions but industrial applications are emerging for electricity producing glazing and ambient light harvesting to power consumer electronic devices and sensors. The best laboratory scale DSC has achieved a PCE of 13.0% certified by an accredited PV testing laboratory, Fraunhofer Institute for Solar Energy Systems (FhG-ISE) in Freiburg, Germany. The PCE value is presented in NREL’s Best Research-Cell Efficiencies Chart. The progress has profited from the development of new sensititzers^1–10^, electrolytes^11–15^, and device structures^16^. DSCs have advantages of offering bifacial light harvesting, semitransparency, esthetically pleasing appearance, and cost-effective fabrication, making them more suitable for distributing power applications than silicon PV^17^. DSC panels have been deployed as demonstration projects for outdoor applications. They have been installed on the façade of SwissTech Convention Center in 2014^7^ and on top of Science Tower of Graz in 2017^18^. Recently, DSCs were found to exhibit an outstanding feature of impressive PCEs over 30% under indoor lighting, superior to amorphous silicon and other thin film semiconductor PV technologies^16^. Thus, they are very promising to serve as power sources to charge portable consumer electronics and sensors. This scenario is being played out, e.g., by the Swedish company Exeger, which is producing flexible DSCs integrated into tablets or earphones to cover their entire energy consumption (https://exeger.com/).

To secure the future successful commercialization of DSCs, further improving their PCE and the durability is warranted. Co-sensitization is one of promising strategies to improve the device performance, especially the short-circuit photocurrent (*J*_sc_) when the dyes have complementary absorption spectra^9,19^. The concept of co-sensitization goes beyond the development of dyes with high molar extinction coefficients and panchromatic absorption. Recent developments of highly efficient DSCs^16,18,20,21^ employ a combination of dyes, one being responsive over a wide spectrum, yielding a large *J*_sc_, but lower *V*_oc_, and a co-sensitizer that absorbs mainly blue and yellow light over a narrower spectrum producing a high open-circuit photovoltage (*V*_oc_) but a low *J*_sc_. Judicious molecular engineering of the two dyes produces a synergistic effect. Benefiting from the large *J*_sc_ of the one sensitizer and the high *V*_oc_ of the other, these photosystems reach a higher PCE and stability than the ones employing either one of dyes^16,18^. In this regard, the development of co-sensitizer with a high *V*_oc_ offers a promising way to augment the performance of DSCs. Recent studies have shown that some organic dyes together with Cu(II/I) redox mediators are able to achieve a *V*_oc_ exceeding 1.0 V^15,16,20,22–26^, due to the lower energy loss and more positive redox potential of electrolytes employing Cu(II/I) complexes compared to iodide/triiodide or cobalt(III/II) complexes^12,15,27^. Co-sensitized solar cells based on [Cu^(II/I)^(tmby)_2_][TFSI]_2/1_ redox electrolyte (tmby = 4,4′,6,6′-tetramethyl-2,2′-bipyridine; TFSI = bis(trifluoromethylsulfonyl)imide) have achieved impressive PCEs, both under full sunlight and indoor lighting conditions^16,26^.

**Fig. 1:**
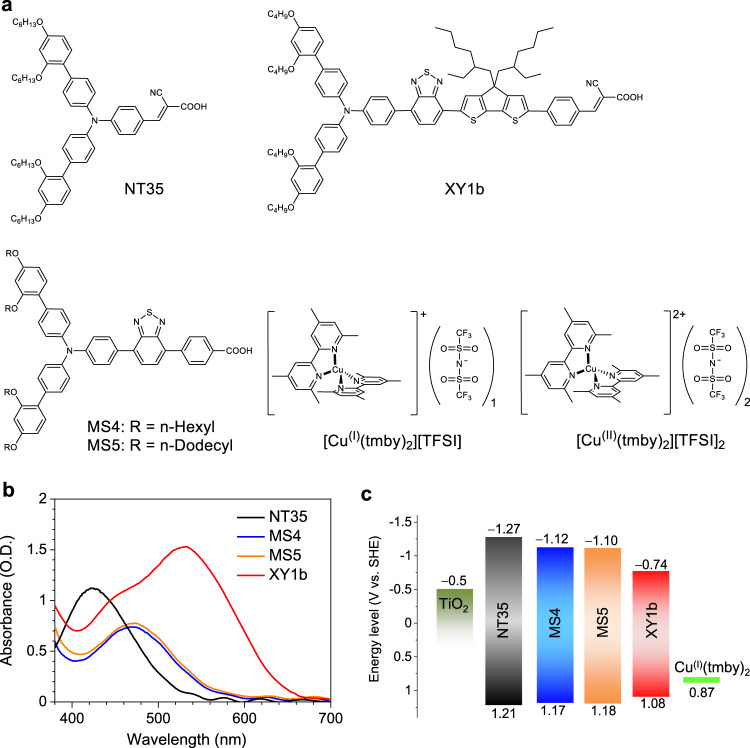
Dye molecular structures and their optical and electronic properties. **a** Molecular structures of dyes (NT35, MS4, MS5, and XY1b) and copper complex ([Cu^(I)^(tmby)_2_][TFSI] and [Cu^(II)^(tmby)_2_][TFSI]_2_, tmby = 4,4′,6,6′-tetramethyl-2,2′-bipyridine; TFSI = bis(trifluoromethylsulfonyl)imide). **b** UV–Vis absorption spectra of NT35, MS4, MS5, and XY1b adsorbed on 2.2 μm thick transparent TiO_2_ films. **c** Energy levels diagram of TiO_2_, dyes, and [Cu^(I)^tmby)_2_][TFSI].

Here, we show the judiciously designed organic donor-acceptor co-sensitizers, coded MS4 and MS5 (Fig. 1a), featuring the bulky donor *N*-(2’,4’-bis(dodecyloxy)-[1,1’-biphenyl]-4-yl)-2’,4’-bis(dodecyloxy)-*N*-phenyl-[1,1’-biphenyl]-4-amine, coined below Hagfeldt donor, and the electron acceptor 4-(benzo[*c*][1,2,5]thiadiazol-4-yl)benzoic acid (BTBA) for high *V*_oc_ in DSCs with the [Cu^(II/I)^(tmby)_2_][TFSI]_2/1_ redox electrolyte. Compared to the reference dye NT35 employing the Hagfeldt donor and cyanoacrylic acid (CA)^28^ as electron acceptor, MS4 with the same donor but BTBA as acceptor has lower interfacial charge recombination rates and higher *V*_oc_. By further elongating the end chains of the Hagfeldt donor of MS4 from *n*-hexyloxy to *n*-dodecyloxy, the resulting MS5 further suppresses the interfacial charge recombination enabling *V*_oc_ of 1.24 V, a record among copper-based DSCs. MS5 has a *V*_oc_ deficit as low as 130 mV and an ideality factor of merely 1.08, which is the lowest value reported for DSCs. The co-sensitization of the high-*V*_oc_ MS5 with a wide spectral-response dye XY1b shows an impressive PCE of 13.5% under standard AM1.5G sunlight condition and remains stable for 1000 h under light soaking conditions. Remarkably, a MS5 + XY1b co-sensitized DSC with an active area of 2.8 cm^2^ achieves a record PCE of 34.5% under ambient light at 1000 lux, with a notable *V*_oc_ of 0.98 V and power output of 109.8 µW cm^−2^.

## Results

### Synthesis and opto-electronic properties of photosensitizers

The synthetic routes of MS4 and MS5 are depicted in the Supplementary Information (SI). Figure 1b shows the UV–Vis absorption spectra of these dyes adsorbed on 2.2 µm thick transparent titanium dioxide (TiO_2_) films in ambient air. The corresponding maximum absorption wavelength is tabulated in Supplementary Table 1. Compared to NT35 with the CA electron acceptor, MS4 with the BTBA electron acceptor shows a 46 nm red shift of maximum absorption wavelength at 468 nm. Substituting the *n*-hexyloxy chains of Hagfeldt donor in MS4 with *n*-dodecyloxy groups, yields the MS5 dye, which has a similar absorption spectrum to MS4. Although the maximal molar extinction coefficient of NT35 within 400−500 nm is 4.9-fold and 3.1-fold higher than that of MS4 and MS5 (Supplementary Fig. 1 and Supplementary Table 1), respectively, the maximal absorbance of NT35 on TiO_2_ is merely 1.5-fold larger than these of both dyes. This result indicates that NT35 has a lower molecular packing density on TiO_2_ film. The measured dye-loading amount of NT35 in TiO_2_ film is 2.6 × 10^−9^ mol cm^−2^ µm^−1^, which is smaller than these of MS4 and MS5 being 5.3 × 10^−9^ mol m^−2^ µm^−1^ and 6.9 × 10^−9^ mol cm^−2^ µm^−1^, respectively.

We performed cyclic voltammetry (CV) measurements of the three dyes adsorbed on TiO_2_ in a three-electrode electrochemical cell to depict their energy level alignments, as shown in Fig. 1c. The CV curves are presented in Supplementary Fig. 2 and the data are tabulated in Supplementary Table 1. The first oxidation potentials (*E*_ox_) of NT35, MS4, and MS5 on TiO_2_ film are 1.21, 1.17, and 1.18 V vs. the standard hydrogen electrode (SHE), respectively, being more positive than the redox potential of [Cu^(I)^(tmby)_2_]TFSI (0.87 V vs. SHE)^15^, which provides a sufficient driving force for dye regeneration. The zero–zero transition energies (*E*_0−0_) estimated from the onset absorption wavelength of dyes on TiO_2_ films (Fig. 1b), are 2.48, 2.29, and 2.28 eV for NT35, MS4, and MS5, respectively. This indicates that the BTBA unit has a stronger electron withdrawing ability than CA, leading to a narrower energy gap for MS4 and MS5 than NT35. The reduction potentials (*E*_red_) of these dyes, defined as *E*_red_ = *E*_ox_ − *E*_0–0_, are estimated to be −1.27, −1.12, and −1.10 V vs. SHE for NT35, MS4, and MS5 (Supplementary Table 1), respectively, being more negative than the conduction band edge of TiO_2_ electrode (ca. −0.5 V vs. SHE)^29^, which ensures sufficient driving force for the electron injection.

### Photovoltaic properties of high *V*_oc_ DSCs with copper electrolyte

**Fig. 2:**
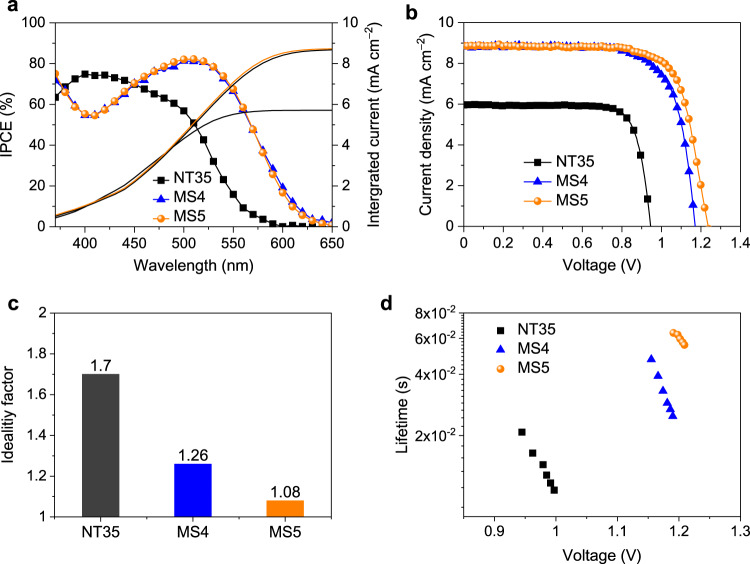
Photovoltaic performance and interfacial charge recombination of co-sensitizers. **a** Incident photon-to-electron conversion efficiency (IPCE) of the dye-sensitized solar cells (DSCs) based on the co-sensitizers of NT35, MS4, and MS5. The solid lines show the corresponding integrated photocurrent calculated from the IPCE. **b** Current density–voltage curves of the DSCs with NT35, MS4, and MS5 measured under AM1.5G, 100 mW cm^−2^ condition. **c** The ideality factors of the DSCs based on the dyes NT35, MS4, and MS5. **d** Comparison of electron lifetimes measured with the small-pulse transient photovoltage decay method against voltage.

DSCs based on the three sensitizers in conjunction with the [Cu^(II/I)^(tmby)_2_][TFSI]_2/1_ electrolyte were fabricated and measured according to the description in Methods. The electrolyte for devices performing under AM1.5 G sunlight conditions is composed of 0.2 M [Cu^(I)^(tmby)_2_]TFSI and 0.1 M [Cu^(II)^(tmby)_2_](TFSI)_2_ with 0.1 M lithium bis(trifuoromethanesulfonyl)imide (LiTFSI) and 0.6 M *N*-methylbenzimidazole (NMB) in acetonitrile. Figure 2a shows the incident photon-to-electron conversion efficiencies (IPCEs) as a function of wavelengths of incident monochromatic lights. The IPCE is determined by the efficiencies of light harvesting, the charge injection, the charge regeneration, and the charge collection. MS4 and MS5 have ∼50 nm red shift of the onset wavelength of IPCE with respect to NT35, and they show maximum IPCE values over 82% at 510 nm, superior to NT35 (75% at 400 nm). Note that the maximum absorbance of MS4 and MS5 on TiO_2_ film is lower than that of NT35 within 400–500 nm (Fig. 1b). The favorable energy level offsets (Fig. 1c) at the interface of dye/TiO_2_ and dye/electrolyte can lead to efficient yields of charge injection and regeneration. The higher IPCE peak of MS4 and MS5 than that of NT35 indicates a more efficient charge collection yield. As evidenced by the following transient photovoltage decay measurement, the devices with MS4 and MS5 have longer electron lifetimes than the one with NT35. The current density–voltage (*J*−*V*) curves measured at an irradiance of simulated AM1.5 G sunlight, 100 mW cm^−2^, are illustrated in Fig. 2b, and the photovoltaic parameters are tabulated in Table 1. The reference dye NT35 has a *V*_oc_ of 0.95 V, a *J*_sc_ of 5.96 mA cm^−2^, a fill factor (FF) of 0.791, and a PCE of 4.5%. MS4 featuring a BTBA acceptor instead of CA exhibits a notably higher *V*_oc_ of 1.17 V, a *J*_sc_ of 8.86 mA cm^−2^, and FF of 0.73, yielding a PCE of 7.6%. By further lengthening the alkyl chains on the Hagfeldt donor from *n-*hexyloxy (MS4) to *n-*dodecyloxy (MS5), the PCE increases further to 8.0% (*J*_sc_ of 8.87 mA cm^−2^, and the FF of 0.73). Remarkably MS5 achieves a strikingly high *V*_oc_ value of 1.24 V, setting a new benchmark for copper-based DSCs. For the given set of components used in this work (dye, oxide, electrolyte), the maximum *V*_oc_ in a DSC is determined by the difference between quasi-Fermi level of TiO_2_, which under intense illumination approaches the energy level of the conduction band edge, and the redox potential of the copper complex^30^. The maximum *V*_oc_ of [Cu^(II/I)^(tmby)_2_][TFSI]_2/1_ electrolyte-based DSC is estimated to be 1.37 V. Thus, the *V*_oc_ deficit of the DSC with MS5 and [Cu^(II/I)^(tmby)_2_][TFSI]_2/1_ is merely 130 mV, indicating a strongly reduced energy loss by interfacial charge recombination.

**Table 1:** Photovoltaic parameters of DSCs measured under standard AM1.5G sunlight.

Dyes	${\boldsymbol{J}}_{{\mathbf{sc}}}^{{\mathbf{IPCE}}}$^a^ (mA cm^-2^)	*J*_sc_^b^ (mA cm^-2^)	*V*_oc_ (V)	FF (%)	PCE (%)
NT35	5.71 ± 0.15	5.96 ± 0.13	0.95 ± 0.004	79.1 ± 0.4	4.5 ± 0.1
MS4	8.69 ± 0.14	8.86 ± 0.15	1.17 ± 0.002	73.0 ± 0.3	7.6 ± 0.1
MS5	8.73 ± 0.18	8.87 ± 0.21	1.24 ± 0.003	73.3 ± 0.4	8.0 ± 0.3
XY1b	15.02 ± 0.15	15.26 ± 0.18	1.01 ± 0.003	76.3 ± 0.2	11.8 ± 0.2
MS5+XY1b	15.41 ± 0.21	15.84 ± 0.24	1.05 ± 0.002	81.3 ± 0.2	13.5 ± 0.2

^a^$J_{{\mathrm{sc}}}^{{\mathrm{IPCE}}}$ values were derived from IPCE spectra. ^b^*J*_sc_ values were experimental values determined from *J*−*V* curves measured under standard AM1.5G, 100 mW cm^−2^ sunlight.

By fitting the *V*_oc_ dependance on the light intensity (*ϕ*) in Supplementary Fig. 3, the ideality factor (*n*) can be obtained via Eq. (1)

1 $$n = \frac{q}{{k_{\mathrm{B}}T}}\frac{{{\mathrm{d}}V_{{\mathrm{oc}}}}}{{{\mathrm{d}}{\mathrm{ln}}(\phi )}}$$

where *q* is the elementary charge, *k*_B_ is Boltzmann’s constant, and *ϕ* is incident photon-flux density. *n* relates to an ideal (*n* = 1) and non-ideal (*n* > 1) diode. For high-quality inorganic semiconductor solar cells, the direct band-to-band radiative charge recombination gives *n* ≈ 1. For organic solar cells, *n* generally departs from unity due to various recombination paths inside band-to-band transitions, such as trap assisted charge recombination. As shown in Fig. 2c, the MS5-based DSC has the lowest *n* of 1.08 compared to the MS4 and NT35-based counterparts, which is also the lowest value in reported literatures of DSCs. The low value of *n* of MS5 indicates largely suppressed interfacial charge recombination from trap states.

We further performed charge extraction and transient photovoltage decay measurements to investigate the interfacial energetics and dynamic properties^31,32^. As shown in Supplementary Fig. 4, at a certain photovoltage an almost invariable amount of charge can be extracted from TiO_2_ films for all cells, implying that the change of dyes may not alter the conduction band edge and electron trap states of TiO_2_. At a given density of extracted charge, the electron lifetimes of MS4-based devices are about two orders of magnitude longer than these of NT35-based counterpart (Fig. 2d). As mentioned above, MS4 has a higher dye-loading on TiO_2_ film compared to NT35 that potentially depresses interfacial charge recombination. Compared to MS4, the electron lifetimes in the MS5 are about 3-times longer, partly due to the slightly higher dye-loading amount of MS5 and longer alkyloxy chain on the Hagfeldt donor to hinder the approach of Cu(II) to the TiO_2_ surface to recombine.

### Highly efficient co-sensitized solar cells under AM1.5G sunlight

**Fig. 3:**
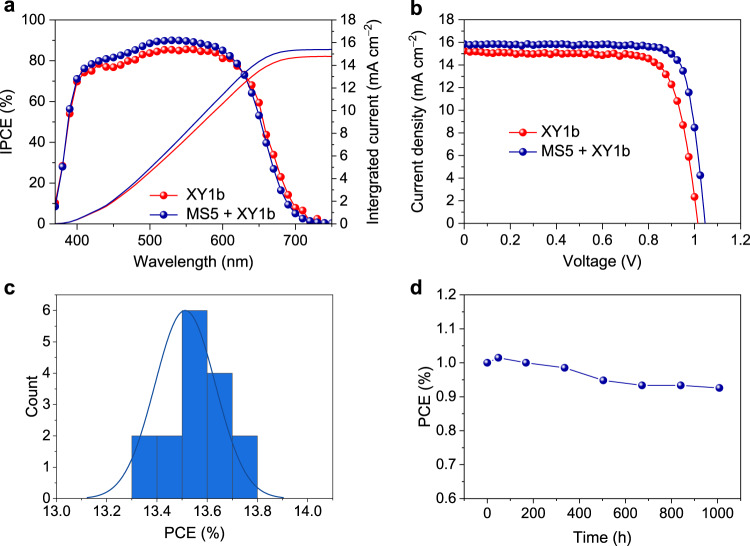
Photovoltaic performance of solar cells based on XY1b and MS5+XY1b. **a** Incident photon-to-electron conversion efficiency (IPCE) of the dye-sensitized solar cells (DSCs) based on XY1b and the co-sensitization of MS5+XY1b. The solid lines show the corresponding integrated photocurrent calculated from the IPCE. **b** Current density–voltage curves of the DSCs with XY1b and the co-sensitization of MS5 + XY1b measured under AM1.5G, 100 mW cm^−2^ condition. **c** Histogram of power conversion efficiency (PCE) of the DSCs based on co-sensitization of MS5+XY1b (16 samples). **d** Evolution of PCE of the DSCs based on MS5+XY1b measured under AM1.5G sunlight (100 mW cm^−2^) during continuous light soaking at 45 °C for 1000 h.

Benefiting from its high *V*_oc_ and strong capacity of retarding interfacial charge recombination, MS5 presents promising features to serve as a co-sensitizer to realize highly efficient DSCs. Herein, we employ MS5 as a co-sensitizer together with the previously reported wide spectral-response dye XY1b^16^ (Fig. 1a). The absorption spectrum of XY1b adsorbed on a mesoscopic TiO_2_ film is shown in Fig. 1b. XY1b has a strong absorption peak at 531 nm, which is red shifted by 63 nm compared to the absorption peak of MS5 within 400−700 nm. The IPCE spectrum of the MS5 + XY1b-based DSC attains slightly higher values than that of XY1b-based counterpart, as shown in Fig. 3a. Figure 3b shows the *J*−*V* curves measured under simulated AM1.5 G 100 mW cm^−2^ sunlight conditions. The control DSC based on XY1b alone shows a PCE of 11.8%, with a *J*_sc_ of 15.26 mA cm^−2^, a *V*_oc_ of 1.01 V, and a FF of 0.763. The performance is similar to our previous result^16^. By contrast, the MS5 + XY1b co-sensitized solar cell exhibits an enhanced *V*_oc_ of 1.05 V, a slightly higher *J*_sc_ of 15.84 mA cm^−2^, which is in good accordance with the integrated current density calculated from IPCE (Fig. 3a and Table 1), and a remarkable FF of 0.813, overall delivering a benchmark PCE of 13.5% (Supplementary Table 2). The PCE histogram of the MS5 + XY1b cells (Fig. 3c) demonstrates the good reproducibility of our results. As shown in Fig. 3d, MS5 + XY1b-based DSC exhibits excellent photostability, maintaining 93% of its initial value during light soaking at full solar intensity (100 mW cm^−2^) at 45 °C for 1000 h. The detailed evolutions of photovoltaic metrics are presented in Supplementary Fig. 5.

### Analysis of the loss of photovoltaic performances under AM1.5G sunlight

**Fig. 4:**
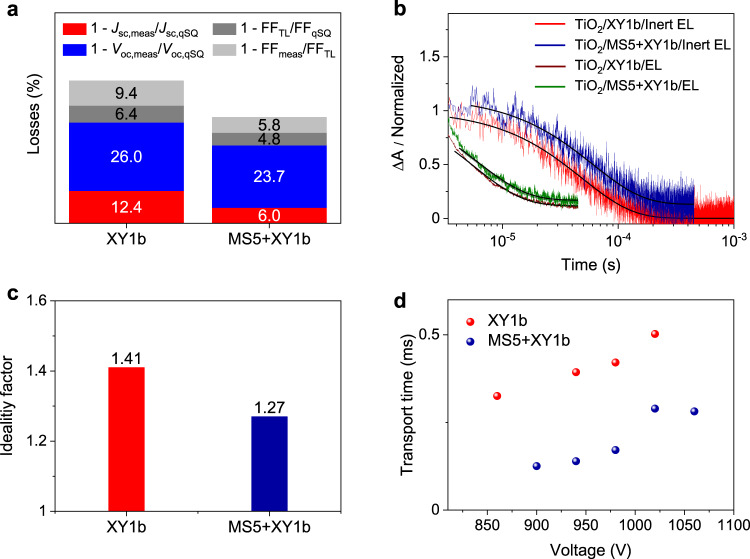
The loss mechanism of photovoltaic parameters of XY1b and MS5 + XY1b. **a** Photovoltaic performance losses due to non-ideal absorption (red), non-radiative recombination (blue and dark gray) and charge transport (light gray). The subscripts stand for measured current density–voltage (*J*−*V*) curve (meas), quasi Shockley–Queisser limit (qSQ), and transport limit (TL). *V*_oc_ and FF stand for open-circuit photovoltage and fill factor, respectively. **b** Nano-second Flash photolysis measurements of dyes-sensitized TiO_2_ films immersed in a [Cu^(II/I)^(tmby)_2_][TFSI]_2/1_ redox electrolyte (EL) or an inert electrolyte (tmby = 4,4′,6,6′-tetramethyl-2,2′-bipyridine; TFSI = bis(trifluoromethylsulfonyl)imide). The solid lines are monoexponential fittings. Pump wavelength: 520 nm; probe wavelength, 815 nm. **c** The ideality factors of the DSCs based on the dyes XY1b and MS5 + XY1b. **d** The transport time as a function of applied voltage.

To unravel the fundamental reasons for the substantial improvement of the performance by co-sensitization, we studied the major PCE loss mechanisms of the XY1b and MS5 + XY1b-based DSCs by analyzing the *J*−*V* curves (Supplementary Fig. 6) following a method inspired by Guillemoles et al. and Stolterfoht et al.^33,34^. The performance losses of *J*_sc_, *V*_oc_, and FF are summarized in Fig. 4a. For the determination of these losses we start with the calculation of a *J*−*V* curve for the DSC-equivalent of the Shockley–Queisser limit, which we call quasi-Shockley–Queisser (qSQ) limit in the following (see Supplementary Fig. 6, red curves). For the qSQ limit of *V*_oc_ we take the difference between the Fermi level of TiO_2_ and the redox potential of the copper complex yielding *V*_oc,qSQ_ = 1.37 V, which represents the maximum *V*_oc_ that can be achieved with this DSC as mentioned above. The qSQ limit of *J*_sc_ is calculated assuming complete solar light absorption, i.e., 100% IPCE, above the absorption onset energy *E*_o_. The latter is determined from the inflection point of the IPCE spectra^35^. The qSQ *J*−*V* curve is then calculated from *V*_oc,qSQ_ and *J*_sc,qSQ_ using Eq. (2)^34^

2 $$J\left( V \right) = J_{0,{\mathrm{qSQ}}}\left( {e^{aV/nk_{\mathrm{B}}T - 1}} \right) - J_{{\mathrm{sc,qSQ}}}$$

with the dark emission current $J_{0,{\mathrm{qSQ}}}$ being determined from the relation $0 = J_{0,{\mathrm{qSQ}}}\left( {e^{qV_{{\mathrm{oc}},{\mathrm{qSQ}}}/nk_{\mathrm{B}}T} - 1} \right) - J_{{\mathrm{sc}},{\mathrm{qSQ}}}$, where *q* is the elementary charge, *k*_B_ the Boltzmann constant and *T* = 298 K the cell temperature (see Supplementary Fig. 6 and Supplementary Table 3). The ideality factor *n* is taken as 1.

As shown in Fig. 4a, the *J*_sc_ loss of MS5 + XY1b (6%) is only half as big as the value for XY1b (12.4%). Although the MS5 + XY1b-based DSC has a slightly blue shifted onset wavelength for the photocurrent response and light-harvesting efficiency spectrum (Supplementary Fig. 7) with respect to XY1b, the higher IPCE for MS5 + XY1b contribute to its smaller *J*_sc_ losses. To reveal the reasons for the smaller *J*_sc_ loss in the co-sensitized solar cell, we employed nano-second laser flash photolysis technique^36,37^ to study the dual-path charge transfer kinetics of the oxidized dye molecules (D^+^) being reduced by either Cu(I) ions in the electrolyte or by recapturing the injected electrons in TiO_2_. As shown in Fig. 4b, when the dye-sensitized TiO_2_ films are in contact with an inert electrolyte consisting of 0.1 M LiTFSI and 0.6 M NMB in acetonitrile, the transient absorption decay signals reflecting the back-electron-transfer from the conduction band of TiO_2_ to D^+^ have a time constant of 49.0 µs and 61.0 µs for XY1b and MS5 + XY1b, respectively. When the dye-sensitized TiO_2_ films are contacted by the [Cu^(II/I)^(tmby)_2_][TFSI]_2/1_ electrolyte, the decays, reflecting the regeneration of D^+^ by [Cu^(I)^(tmby)_2_]TFSI, display lifetimes that are one order of magnitude shorter (5.7 and 6.0 µs for XY1b and MS5 + XY1b, respectively). Overall, the calculated dye regeneration efficiencies are around 86.4% and 91.0% for XY1b and MS5 + XY1b, respectively, which partly explains the higher IPCE plateaus and lower *J*_sc_ losses of co-sensitized solar cells.

The *V*_oc_ losses are 26.0% and 23.7% for XY1b and MS5 + XY1b, respectively. The *V*_oc_ losses are due to residual non-radiative recombination at the TiO_2_/electrolyte interface. The charge extraction and transient photovoltage decay measurements show that the MS5 + XY1b impregnated TiO_2_ film has a threefold longer electron lifetime than that sensitized by XY1b alone (Supplementary Fig. 8), which proves that co-sensitization is effective in retarding the interfacial charge recombination. Note also the higher dye-loading amounting to 2.70 × 10^−8^ mol cm^−2^ μm^−1^ for MS5 + XY1b (XY1b: 1.77 × 10^−8^ mol cm^−2^ μm^−1^ and MS5: 0.93 × 10^−8^ mol cm^−2^ μm^−1^) compared to only 1.97 × 10^−8^ mol cm^−2^ μm^−1^ for XY1b alone, which retards the recombination of conduction band electrons in TiO_2_ with copper(II) ions in the electrolyte.

We evaluated the contribution from non-radiative recombination by calculating a *J*−*V* curve from the diode equation using the measured *J*_sc_, *V*_oc_, and ideality factor *n*. We determined *n* from the light intensity dependent *V*_oc_ measurement of the DSCs (Supplementary Fig. 9). As shown in Fig. 4c, MS5 + XY1b sensitized DSC has a smaller *n* of 1.27 than the value *n* = 1.41 derived for XY1b. Since this calculated *J*−*V* curve (blue curves in Supplementary Fig. 6) takes into account non-radiative recombination losses but neglects losses stemming from non-ideal charge transport, the FF transport limit (FF_TL_) of this transport limit *J*−*V* curve indicates the contribution from non-radiative recombination. The MS5 + XY1b cell has a substantially smaller loss of FF_TL_ (4.8%) than the XY1b cell (FF_TL_ = 6.4%), indicating a suppression of non-radiative recombination in the co-sensitized system. In a real device, charge transport losses manifest themselves in a further reduction in FF.

The FF losses of the measured *J*−*V* curve with respect to FF_TL_ are 9.4% and 5.8% for XY1b and MS5 + XY1b-based DSCs, respectively, indicating a smaller transport loss in co-sensitized system. To scrutinize the reason for the difference of the transport losses between XY1b and MS5 + XY1b, electrochemical impedance spectroscopy (EIS) measurements of devices under white light LED illumination with 100 mW cm^−2^ intensity were performed. The measured spectra were fitted to the data by employing the ZView software and the transmission-line model^38^. As shown in Fig. 4d, the transport time of the MS5 + XY1b-based DSC is shorter than that of XY1b alone based counterpart, which indicates a faster electron transport through the mesoscopic TiO_2_ layer to the external circuit and more efficient charge collection, contributing to its smaller transport loss. In addition, the smaller Warburg diffusion resistance for the MS5 + XY1b-based DSC with respect to that of XY1b counterpart (Supplementary Fig. 10) contributes to its smaller transport loss. The detailed molecular origin of this observation is not clear and beyond the scope of this work.

### Highly efficient co-sensitized solar under ambient lighting

**Fig. 5:**
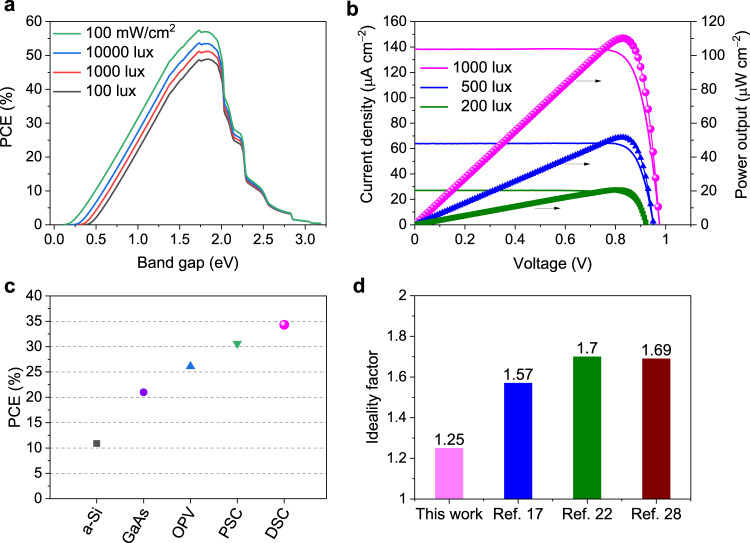
The theoretical maximal PCE and the photovoltaic performance of MS5 + XY1b-based DSC under indoor lighting. **a** Band gap dependent simulations of the theoretical power conversion efficiency (PCE) limitations (Shockley–Queisser limit) for different intensities of the model Osram 930 Warm White fluorescent tube light. **b** Current density–voltage curves and power output of the dye-sensitized solar cell (DSC) with a photoactive area of 2.80 cm^2^ under different ambient light intensities. **c** Summary of the best indoor PCEs of various types of solar cells with the photoactive area of at least 1 cm^2^, including amorphous silicon (a-Si), GaAs, organic polymers (OPV), metal halide perovskite (PSC), and DSC. **d** Summarized the ideality factors of representative publications on DSCs under ambient light.

The DSC is a unique photovoltaic technique that has achieved impressive PCE under ambient light. Different from direct solar radiation, only a relatively small number of electrons can be generated under the lower incident power of ambient light compared to full sun illumination. Therefore, the suppression of recombination processes plays a crucial role in the DSC performance under diffuse light. The above results imply that the DSC based on MS5 + XY1b can suppress the charge recombination very well, and the ideality factor close to one implies a low level of non-radiative losses that there is a small *V*_oc_ decrement with decreasing light intensity. In this regard, a DSC based on MS5 + XY1b with an active area of 2.8 cm^2^ was fabricated to investigate its performance under ambient lighting using a standard Osram 930 Warm White fluorescent tube as light source. Depending on the energy gap of a light absorbing material under different intensities of the model Osram 930 Warm White fluorescent tube light, the theoretical PCE limitations (Shockley–Queisser limit) are calculated with etaOpt software^39^, as shown in Fig. 5a. Owing to the narrow band distribution of the spectra (Supplementary Fig. 11), the energy gap of 1.73 eV yields a theoretical maximum PCE of 49%, 51%, 54%, and 57% at the light intensity of 100 lux, 1000 lux, 10000 lux, and 314,465 lux (100 mW cm^−2^) under Osram 930 Warm White fluorescent tube condition, respectively. Note that the energy gap of MS5 + XY1b system is estimated to be 1.72 eV from the onset of the IPCE spectrum, which matches well the optimal band gap for solar cells working under the Osram 930 Warm White fluorescent tube indoor lighting.

The DSCs tested under ambient light employed an electrolyte with lower concentrations of Cu(II) complexes to support the lower photocurrents generated under these conditions by the photovoltaic^17^. As shown in Supplementary Table 4, reducing the concentration of Cu(II) can suppress interfacial charge recombination and improve in particular the *V*_oc_ and overall device performance, under ambient light. We further found that lowering the concentration of Cu(I) also benefited the PCE. Devices tested under the ambient light employed an electrolyte composed of 0.1 M [Cu^(I)^(tmby)_2_]TFSI and 0.04 M [Cu^(II)^(tmby)_2_](TFSI)_2_ complexes together with 0.1 M LiTFSI and 0.6 M NMB in acetonitrile. The *J*−*V* curves of DSCs were recorded at 1000, 500, and 200 lux, as shown in Fig. 5b. The photovoltaic parameters are tabulated in Table 2. At 1000 lux, the DSC achieves an impressively high *V*_oc_ of 0.98 V, exceeding that of all previously reported devices. Along with the short-circuit photocurrent of 387 µA (*J*_sc_ = 138.2 µA cm^−2^) and FF of 0.815, the device produces a maximal power of 307.4 µW (*P*_max_ = 109.8 µW cm^−2^) that corresponds to an unprecedented PCE of 34.5%. Furthermore, DSCs with larger areas of 8.0 cm^2^ and 20.25 cm^2^ achieved PCEs of 31%^26^ and 26%^16^ at 1000 lux, respectively. These results place DSCs in a leading position for ambient light-harvesting applications, surpassing the performance of other types of solar cells made of amorphous silicon (a-Si)^40^, GaAs^20^, organic polymers (OPV)^41^, and metal halide perovskite (PSC)^42^ (Fig. 5c and Supplementary Table 5). At 500 and 200 lux, the *V*_oc_ of the DSC remains over 0.92 V and delivers PCEs of 32−33% (Table 2). As shown in Fig. 5d, the ideality factor *n* of this device is 1.25 under dim light, lower than that of the previous benchmark DSCs^16,20,26^ (Supplementary Fig. 12 and Supplementary Table 6). Remarkably, the DSC retain well its initial PCE after 500 h under continuous illumination under indoor light (Supplementary Fig. 13), reflecting excellent stability.

**Table 2:** Photovoltaic parameters of the DSC based on MS5 + XY1b with photoactive area of 2.80 cm^2^ (length: 40 mm; width: 7 mm) under different light intensities from an Osram 930 Warm White fluorescent light.

Cell performance	*P*_in_ of Osram 930 Warm White Light (lux or µW cm^−2^)		
	1000 (318.2)	500 (159.1)	200 (63.6)
*P*_max_ (µW)	307.4	143.9	57.7
PCE (%)	34.5	32.3	32.4
*V*_oc_ (V)	0.98	0.95	0.92
*I*_sc_ (mA)	0.387	0.179	0.076
FF	0.815	0.845	0.826

## Discussion

In conclusion, we designed and synthesized two simple high-*V*_oc_ organic dyes (MS4 and MS5) through judiciously tailoring the electron acceptor and Hagfeldt donor for copper-based DSC. The dye MS5, characteristic of Hagfeldt donor with *n*-dodecyl chains and the electron acceptor BTBA, yields the highest *V*_oc_ of 1.24 V with the *V*_oc_ deficit as low as 130 mV and ideality factor of 1.08, benefited from a retarded interfacial charge recombination. The co-sensitization of MS5 and XY1b achieves highly efficient and stable DSCs with a remarkable high PCE of 13.5% under standard AM1.5G sunlight condition. Strikingly, under standard Osram 930 Warm White fluorescent tube light at 1000 lux intensity, the DSC with the active area of 2.8 cm^2^ presented an impressive PCE of 34.5% with a distinctive *V*_oc_ of 0.98 V and power outputs of 109.8 µW cm^−2^. Such high PCEs stems from a very low ideality factor, mainly because of the reduced interfacial charge recombination. Our work highlights the importance of judicious molecular engineering of high-*V*_oc_ co-sensitizers to improve photovoltaic performance of DSCs based on copper redox electrolyte.

## Methods

### Materials

Acetonitrile (ABCR), chloroform (CF) (ABCR), *tert*-butanol (Sigma-Aldrich), ethanol (EtOH) (Acros), *N*-methylbenzimidazole (Sigma-Aldrich), chenodeoxycholic acid (Sigma-Aldrich), lithium bis(trifuoromethanesulfonyl)imide (LiTFSI) (Sigma-Aldrich). The powders of [Cu^(I)^(tmby)_2_][TFSI] and [Cu^(II)^(tmby)_2_][TFSI]_2_ were purchased from Dyenamo AB, and used as received without further purification. The powders of XY1b dye molecules were purchased from Dyenamo AB, and further purified before the use. The dye NT35 was synthesized according to the previous^28^. The photosensitizing dye MS4 and MS5 were prepared according to the synthetic routes in the Supplementary Fig. 14.

### Fabrication of solar cells

The mesoporous TiO_2_ electrodes were fabricated following the literature procedure^16^. The Fluorine doped tin oxide (FTO) glass (Nippon Sheet Glass, NSG, 10 ohms sheet resistance) was thoroughly cleaned with water, acetone, and ethanol in sequence. Then the substrates were treated by 50 mM TiCl_4_ aqueous solution at 70 °C for 50 min before a 15 min ultraviolet/O_3_. The TiO_2_ pastes of 18 NR-T or 30 NR-D (Greatcell Solar Limited) and the light-scattering TiO_2_ particles (Greatcell Solar Limited, WER2-0) were sequentially deposited on the above FTO glass by screen-printing technology. The TiO_2_ films were patterned in round spots with the area of 0.28 cm^2^ or rectangular shapes (40 × 7 mm). The mesoporous TiO_2_ films composed of ~4.0 μm transparent layer and ~4.0 μm light-scattering layer were obtained after the films were sintered in dry air flow and gradually cooled down to room temperature. The mesoporous TiO_2_ films for ambient light devices were treated in TiCl_4_ (40 mM) for 40 min in an oven at 70 °C. The mesoporous TiO_2_ films for 1 sun devices were without TiCl_4_ solutions post-treatment. The mesoporous TiO_2_ electrodes were stained by immersing them into dye solutions at room temperature for 14 h before sintered at 500 °C in air for 30 min and cooled down to 80 °C. The dye solutions of NT35, MS4, and MS5 was prepared by dissolving 0.1 mM corresponding dye in acetonitrile/*tert*-butanol (*v*/*v*, 1/1). The dye solution of XY1b was made by dissolving 0.1 mM XY1b and 2.5 mM chenodeoxycholic acid in CF/EtOH (*v*/*v*, 1/9). The solution for co-sensitization of MS5/XY1b was prepared by dissolving 0.05 mM MS5, 0.1 mM XY1b, and 0.5 mM chenodeoxycholic acid in CF/EtOH (*v*/*v*, 1/9). The counter electrode (the PEDOT film coated FTO glass) was prepared using the electrical deposition technique following the literature procedure^16^. Both electrodes were pressed together mechanically without spacer and further sealed with UV light curing glue (ThreeBond 3035B), which is quickly solidified by UV light from a UV curing machine (TEKLITE). The electrolyte was injected into the sealed electrodes through a predrilled hole on the counter electrode to complete the fabrication of the sandwich-type DSC. The hole was sealed with UV light curing glue. All cells for characterizations were sealed. The copper-based electrolyte consisting of 0.2 M [Cu^(I)^(tmby)_2_]TFSI and 0.1 M [Cu^(II)^(tmby)_2_](TFSI)_2_ complexes with 0.1 M LiTFSI and 0.6 M NMB in acetonitrile was used to obtain high efficiency under one sun. The copper-based electrolyte consisting of 0.1 M [Cu^(I)^(tmby)_2_]TFSI and 0.04 M [Cu^(II)^(tmby)_2_](TFSI)_2_ complexes with 0.1 M LiTFSI and 0.6 M NMB in acetonitrile was used to obtain high efficiency under ambient light.

### Characterization of solar cells

During the IPCE and *J*−*V* measurements, the DSCs with a photoactive area of 0.28 cm^2^ were masked with an aperture area of 0.158 cm^2^. The simulated solar light was provided by a 300 W Xenon light source from Oriel, which is equipped with a SchottK113 Tempax sunlight filter (Praezisions Glas & OptikGmbH) to match the emission spectrum of the lamp to the AM1.5G standard. The light intensity was determined using a calibrated Si reference diode equipped with an infrared cutoff filter (KG-3, Schott). The attenuated light intensities of ∼50 and ∼10 mW cm^−2^ were obtained by using the metal mesh. The DSCs with the photoactive areas of 2.80 cm^2^ were masked with rectangular areas of 3.80 cm^2^ and the artificial indoor light was provided by the OSRAM 930 Warm White tube light. The light intensities were calibrated by the light meter (TES-1334, TES). The *J*–*V* curves were recorded by a Keithley 2400 source meter. The voltage scan rate was set to 125 mV s^−1^. IPCE was recorded with a commercial apparatus (Aekeo-Ariadne, Cicci Research s.r.l.) based on a 300-W Xenon lamp. During the measurement, the solar cell was illuminated under the white light at 10 mW cm^−2^ supplied by an array of white LED. For light soaking ageing test at intensity of 100 mW cm^−2^ at 45 °C, the solar cells were stressed under open-circuit conditions. For stability test, the devices with UV light curing glue sealant were further sealed with 3 M™ Scotch Weld™ Epoxy Adhesive DP460 to prevent the electrolyte from leaking and block molecules such as water and oxygen from penetrating into the devices.

### Electrochemical and UV–Vis absorption spectra characterizations

The electrochemical characterization of metal oxide surface-attached dyes was performed on a BioLogic SP300 potentiostats in a classical three-electrode configuration using electrochemical cyclic voltammetry (CV) and differential pulse voltammetry (DPV) procedures. The dye adsorbed on 2.2 µm thick transparent mesoscopic TiO_2_ film was used as working electrode, a graphite rod was used as counter electrode, and a silver wire covered with silver chloride (Ag/AgCl) immersed in a 0.1 M LiTFSI solution in acetonitrile was used as a quasi-reference electrode (QRE) in acetonitrile in a sealed bridge tube. The supporting electrolyte was 0.1 M LiTFSI in dry acetonitrile. The QRE was calibrated against the ferrocene/ferrocenium redox system by cyclic voltammetry. A Perkin-Elmer Lambda 950 spectrophotometer was used to record UV–Vis absorption spectra. The UV–Vis absorption spectra of dyes adsorbed on 2.2 µm thick transparent TiO_2_ films were measured in ambient air.

### Transient photovoltage decay and charge extraction measurements

Electron lifetime and extracted charge measurements were performed using the Dyenamo toolbox using a white LED (Luxeon Star 1 W) as light source. Voltage traces and current traces were recorded with a 16-bit resolution digital acquisition board (National Instruments), then lifetimes and extracted charges were determined by monitoring photovoltage transients at different light intensities upon applying a small square wave modulation to the base light intensity. The photocurrent and photovoltage responses were fitted with using first-order kinetics to obtain time constants.

### Nano-second flash photolysis measurement

The samples were excited at *λ* = 520 nm by 10 ns-duration laser pulses produced by a tunable optical parametric oscillator (OPO-355, GWU) pumped by a frequency-tripled (355 nm), Q-switched Nd:YAG laser (Surelite, Continuum, 20 Hz repetition rate). The excitation light fluence was reduced by use of various neutral density filters to 0.6 mJ cm^−2^ per pulse (for samples with redox-active electrolyte) and 2.2 μJ cm^−2^ per pulse (for samples without redox-active electrolyte). The time evolution of the absorbance change of the sample was probed at *λ* = 815 nm by passing a cw light beam produced by a Xe arc lamp through various IR and cutoff filters and a first monochromator before reaching the sample at an angle of 60°. The probe beam was finally collected by a second grating monochromator (CS260, Newport) and detected by a fast Si avalanche photodiode (APD410A/M, Thorlab). The transient voltage signal was recorded by a fast oscilloscope (DPO 7104C, Tektronix) and averaged over 5000 laser shots. The data were extracted with a MATLAB routine and fitted using a first-order decay Eq. (3)

3 $$f\left( x \right) = y0 + A \ast e^{ - x/\tau }$$

### Electrochemical impedance measurements

Impedance measurements were performed using a BioLogic SP300 potentiostat, over a frequency range from 1 MHz down to 0.1 Hz at bias potentials between 0 and 1.06 V (with a 40-mV sinusoidal AC perturbation). All measurements were done at 25 °C. The resulting impedance spectra were analyzed with Z-view software (v2.8b, Scribner Associates Inc.).

### Reporting summary

Further information on research design is available in the Nature Research Reporting Summary linked to this article.

## Supplementary information

Supplementary Information

Peer Review File

Solar Cells Reporting Summary

## Data Availability

All relevant data in this study are available from the corresponding authors upon request.
