# Supplementary material for: A molecular photosensitizer achieves a Voc of 1.24 V enabling highly efficient and stable dye-sensitized solar cells with copper(II/I)-based electrolyte
Source: Nat Commun. 2021 Mar 19;12:1777. doi: 10.1038/s41467-021-21945-3 (PMC7979847; doi:10.1038/s41467-021-21945-3)
Supplement: Supplementary file 1 — Supplementary Information [file 41467_2021_21945_MOESM1_ESM.pdf]

Supplementary Information

A Molecular Photosensitizer Achieves a V_{oc} of 1.24 V Enabling Highly Efficient and Stable Dye Sensitized Solar Cells with Copper(II/I)-based Electrolyte

Dan Zhang[1,2†], Marko Stojanovic[1†], Yameng Ren[1,†,★], Yiming Cao [1,4], Felix T. Eickemeyer[1,2★], Etienne Socie[3], Nick Vlachopoulos[2], Jacques-E. Moser[3], Shaik M. Zakeeruddin[1], Anders Hagfeldt[2,★], and Michael Grätzel[1,★]

[1]Laboratory of Photonics and Interfaces, Institute of Chemical Sciences & Engineering, École Polytechnique Fédérale de Lausanne, 1015 Lausanne, Switzerland

[2]Laboratory of Photomolecular Science, Institute of Chemical Sciences & Engineering, École Polytechnique Fédérale de Lausanne, 1015 Lausanne, Switzerland

[3]Photochemical Dynamics Group, Institute of Chemical Sciences and Engineering, École Polytechnique Fédérale de Lausanne, 1015 Lausanne, Switzerland

[4]Current address: H.Glass SA, EPFL Innovation Park, 1015 Lausanne, Switzerland

[†]These authors contributed equally to this work

[★]e-mail : yameng.ren@epfl.ch; felix.eickemeyer@epfl.ch; anders.hagfeldt@epfl.ch; michael.graetzel@epfl.ch;

Supplementary Methods

Materials. Ethanol (EtOH) (Acros), methanol (Sigma), dichloromethane (DCM, Thommen-Furler), hexanes (VWR International SA), diethyl ether (HUBERLAB AG), hydrochloric acid (VWR International SA), potassium phosphate tribasic (Acros), magnesium sulfate (Reactolab), sodium tert-butoxide (ABCR), potassium hydroxide (MERCK), acetic acidpotassium salt (Sigma), bis(pinacolato)diboron (Fluorochem), Aniline (Sigma), *N*-bromosuccinimide (NBS, TCI), 4,7-dibromo-2,1,3-benzothiadiazole (Combi-Blocks, Inc.), 4-ethoxycarbonylphenylboronic acid (Fluorochem), 2-(dicyclohexylphosphino)-2',4',6'-triisopropylbiphenyl (Fluorochem), bis(triphenylphosphine)palladium(II) chloride (Fluorochem), tris(dibenzylideneacetone)dipalladium(0) (Sigma), and tris(dibenzylideneacetone)dipalladium(0) (Sigma) were used as received without further purification. Anhydrous THF and Toluene were purchased over molecular sieves from Acros Organics. The solvents used for palladium catalyzed cross coupling reactions were degassed three times using the freeze-pump-thaw method and liquid nitrogen. The photosensitizing dye MS4 and MS5 were prepared according to synthesis of the dyes part in the Supplementary Information.

Characterizations of new compounds. ^{1}H and ^{13}C NMR spectra were recorded on Bruker AvanceIII-400 MHz NMR spectrometer with tetramethylsilane as the internal standard. Chemical shifts are reported in parts per million (ppm, δ). Mass spectra were collected on a HITACHI-80 mass spectrometer.

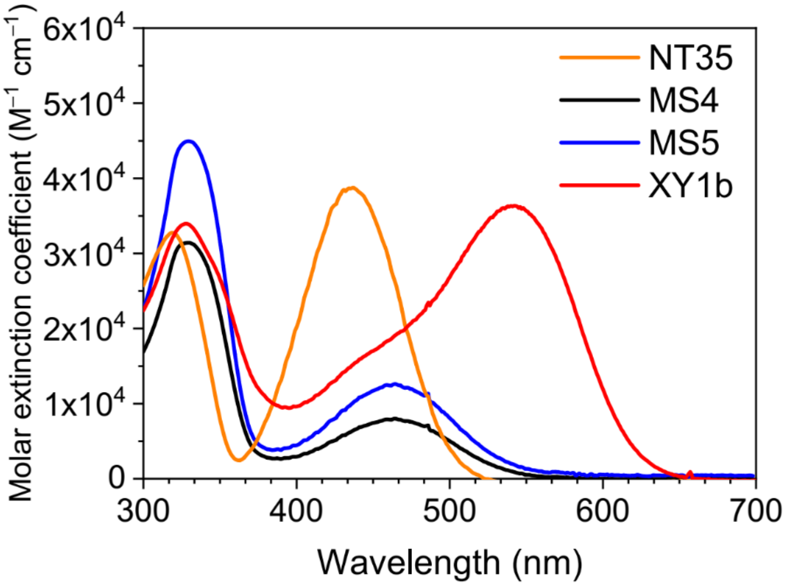

Supplementary Fig. 1 | Absorption spectra of dyes NT35, MS4, MS5, and XY1b dissolved in THF.

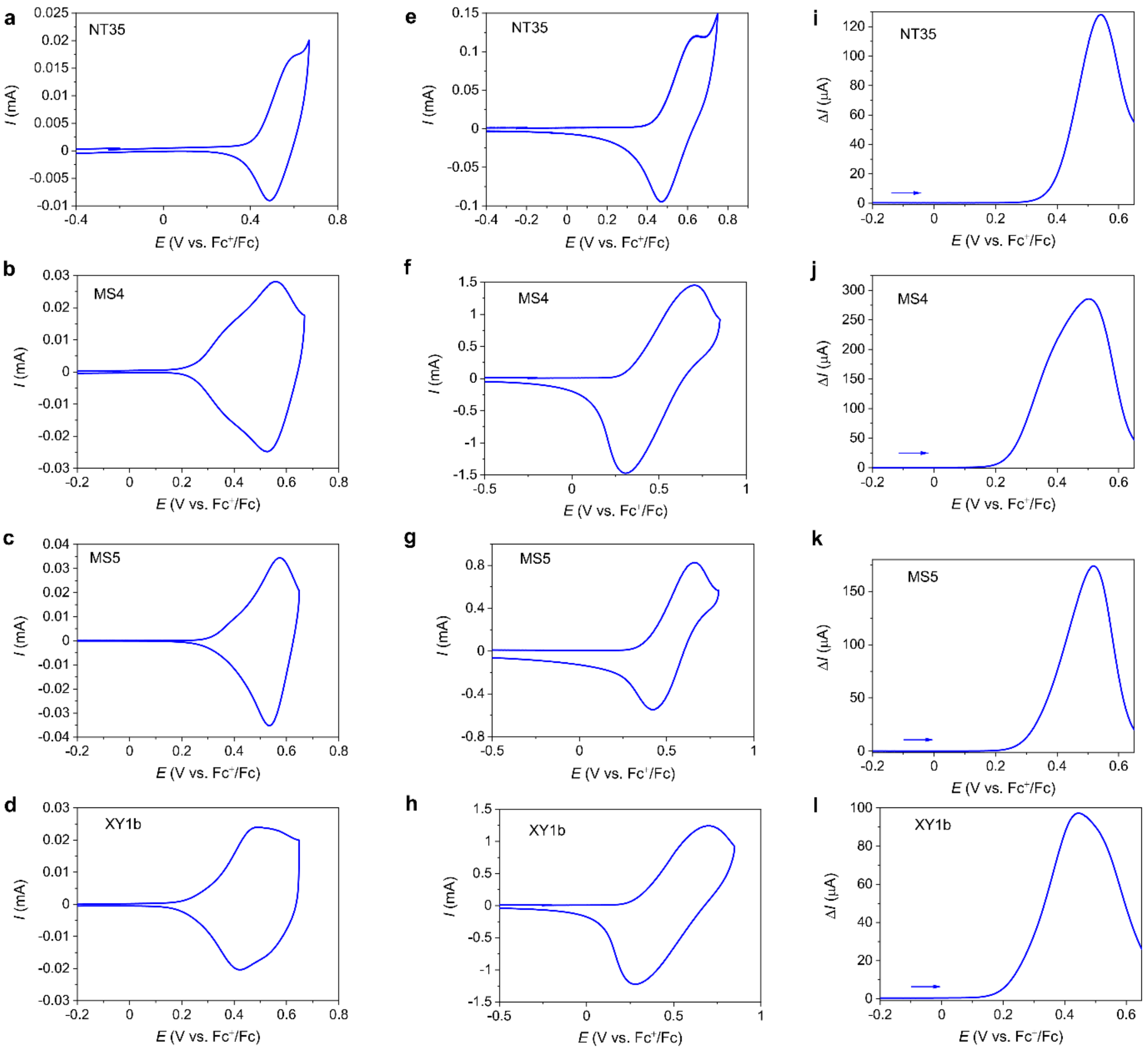

Supplementary Fig. 2 | (a–h) Cyclic voltammogram curves of dyes adsorbed on 2.2 μm thick mesoscopic TiO_2 films supported by FTO electrodes in acetonitrile using a 0.1M LiTFSI solution as supporting electrolyte. The scan rates were 10 mV/s for a–d, 100 mV/s for e, and 1V/s for f–h. (i–l) Differential pulse voltammetry curves of dyes adsorbed on a 2.2 μm thick transparent TiO_2 films/FTO electrodes in a 0.1M LiTFSI solution in acetonitrile as supporting electrolyte at a scan rate (forward) 4 mV/s, pulse height 50 mV, and pulse width 100 ms.

Supplementary Note 1

Cyclic voltammetry (CV) is widely used for determining the standard electrode potential (E^o) for both solution species and surface-attached species. In the case of Nernstian reactions at surface-attached species, the peak potential for both the anodic ($E_{max(an)}$) and the cathodic ($E_{max(cat)}$) branch of the voltammogram is equal to E^o: $E_{max(an)} = E_{max(cat)} = E^o$. The equation applies either for monolayers or for thick layers in which surface diffusion is very fast at the

time scale of the experiment. For quasi-reversible systems, as it is the case for the metal oxide-attached dyes in this work, with slow electrode kinetics, the relation $E_{max(an)} > E^{o} > E_{max(cat)}$ holds. However, even a Nernstian system can appear as quasi-reversible, with $E_{max(an)} - E_{max(cat)} > 0$, in presence of a high Ohmic resistance causing a substantial voltage drop between working and reference electrode due to high electrolyte and electrode resistance (the latter mainly from the conducting FTO glass). A reasonable and more accurate approximation for E^{o} used in the present work is, taking into account the deviation from Nernstian behaviour mostly due to ohmic losses, that using the following Equation (1)[1]:

$$E^{\circ} = E_{\max(\text{cat})} + \left[E_{\max(\text{an})} - E_{\max(\text{cat})}\right] \frac{I_{\max(\text{an})}}{I_{\max(\text{an})} + |I_{\max(\text{cat})}|} \quad (1)$$

where $I_{max(an)}$ and $I_{max(cat)}$ are the anodic and cathodic peak currents. The E^{o} value determined above is the intercept of the line connecting the two peaks with the axis of zero current. From the CV curves in Supplementary Fig. 2 a–d, the calculated E^{o} values are 0.581 V, 0.544 V, 0.555 V, and 0.459 V vs. Fc^{+}/Fc (ferrocenium/ ferrocene) for NT35, MS4, MS5 and XY1b, respectively. These were converted to E^{o} values vs. the standard hydrogen electrode (SHE) by using the conversion factor E° (Fc^{+}/Fc) = 0.624 V/SHE in acetonitrile as proposed by Pavlishchuk and Addison[2] yielding 1.21 V, 1.17 V, 1.18V, and 1.08 V for NT35, MS4, MS5 and XY1b, respectively. These are listed in Supplementary Table 1. CV measurements using a faster scan rate of 1 V/s were also performed, as shown in Supplementary Fig. 2 e–h, which show larger separations of the cathodic and anodic peaks. This reflects the effect of the ohmic resistance of the electrolyte and FTO supported TiO_2 electrode which causes a large separation between the anodic and cathodic peak from E° rendering its measurement less precise. Therefore, we used the values from the CV curves (Supplementary Fig. 2 a–d) at slow scan rate (10 mV/s) in this work. These results are further confirmed by differential pulse voltammetry (DPV) measurements (Supplementary Fig. 2 i–l), the E° values being 0.542 V, 0.504 V, 0.519 V, and 0.445 V vs. Fc^{+}/Fc for NT35, MS4, MS5 and XY1b, respectively.

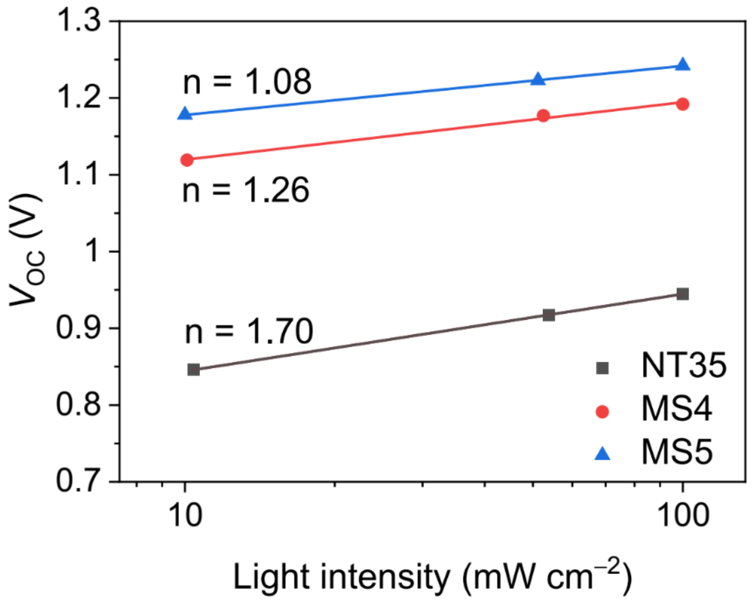

Supplementary Fig. 3 | Open circuit photovoltage (V_{oc}) as a function of different light intensity (ϕ) of the DSCs based on NT35, MS4, and MS5 for the calculation of ideality factor (n).

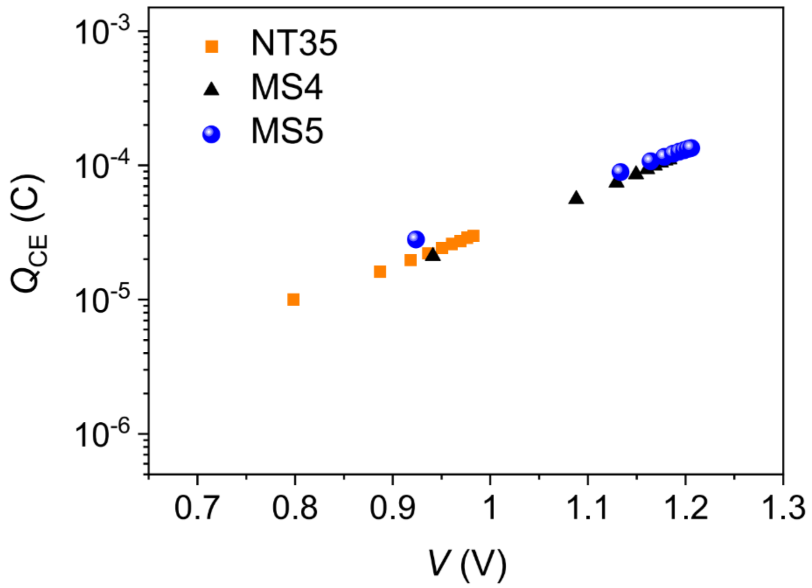

Supplementary Fig. 4 | Plots of charges (Q_{CE}) extracted in a dyes-grafted TiO_2 film measured with charge extraction as a function of open-circuit photovoltage (V_{oc}).

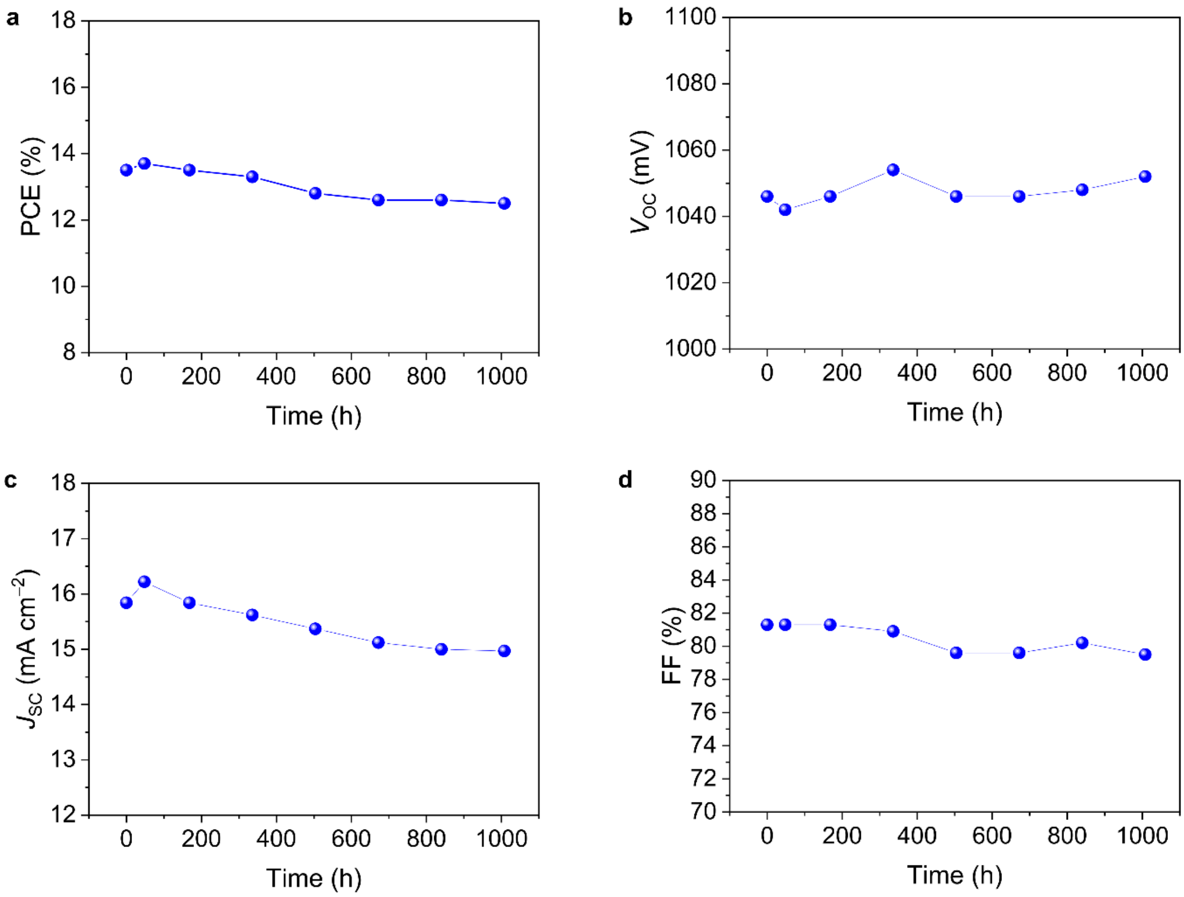

Supplementary Fig. 5 | Evolution of photovoltaic parameters of (a) power conversion efficiency (PCE), (b) open-circuit photovoltage (V_{oc}), (c) short-circuit current density (J_{sc}) and (d) fill factor (FF) of dye sensitized solar cell (DSC) based on co-sensitization of XY1b/MS5 in conjunction with $[Cu^{(II)/(I)}(tmby)_2](TFSI)_{1/2}$ (tmby = 4,4′,6,6′-tetramethyl-2,2′-bipyridine; TFSI = bis(trifluoromethylsulfonyl)imide) redox shuttles measured under standard AM1.5G condition during aging under the full sunlight at 45 °C for 1,000 h.

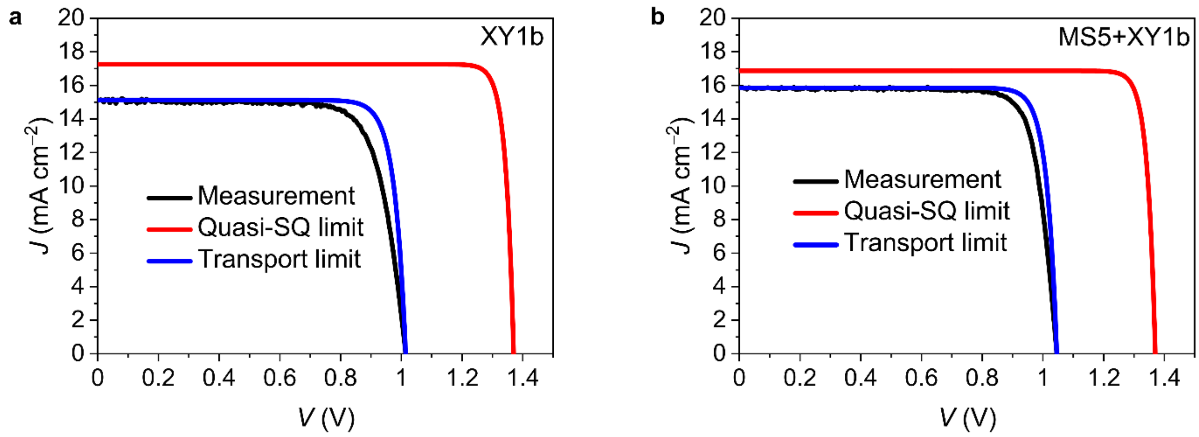

Supplementary Fig. 6 | Current density-voltage (*J*-*V*) curves of the measured device (black curves), quasi-Shockley-Queisser (red curves) and transport limits (blue curves) of the XY1b (a) and MS5+XY1b (b) based DSCs as explained in the main text. The detailed parameters are shown in Supplementary Table 3.

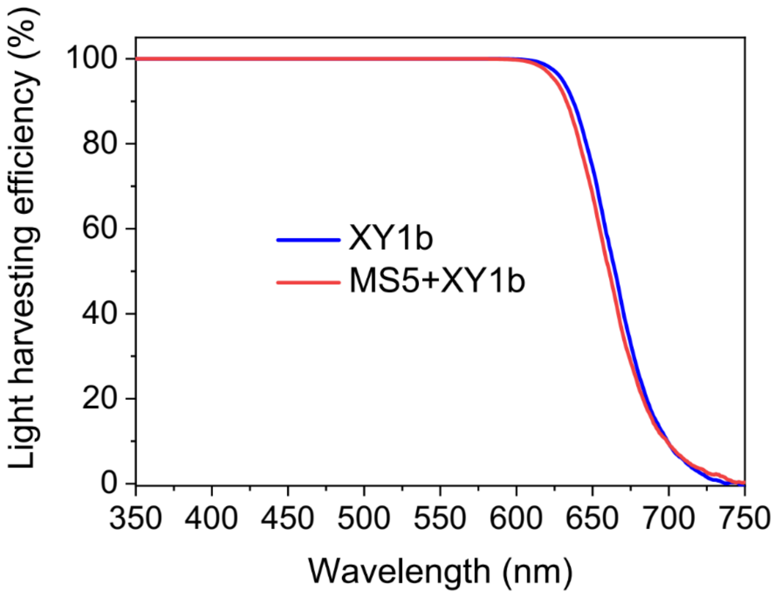

Supplementary Fig. 7 | Light-harvesting efficiency as a function of wavelength for the 8.0-μm-thick dye-sensitized TiO_2 films immersed in a copper-based electrolyte.

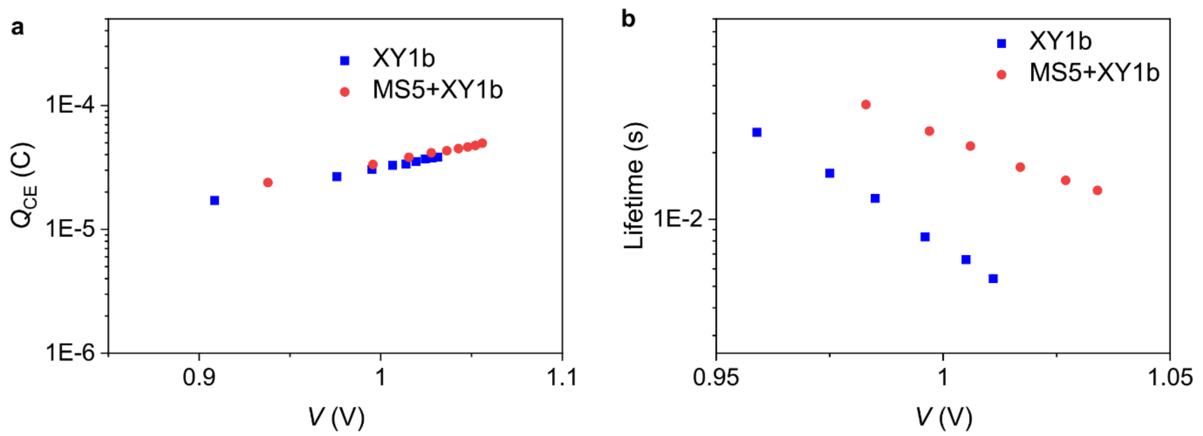

Supplementary Fig. 8 | (a) Plots of charges (Q_{CE}) stored in a dyes-grafted TiO_2 films measured with charge extraction as a function of open-circuit photovoltage (V_{oc}). (b) Comparison of electron lifetimes measured with the small-pulse transient photovoltage decay method against applied voltage (V).

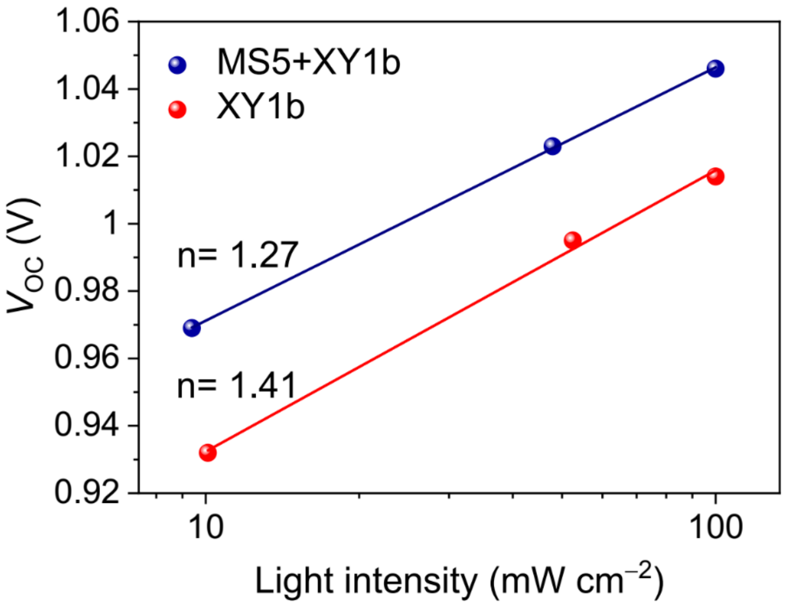

Supplementary Fig. 9 | Open circuit photovoltage (V_{oc}) as a function of different AM1.5G sunlight intensity (ϕ) of the DSCs based on XY1b and co-sensitization of MS5+XY1b for the calculation of ideality factor (n).

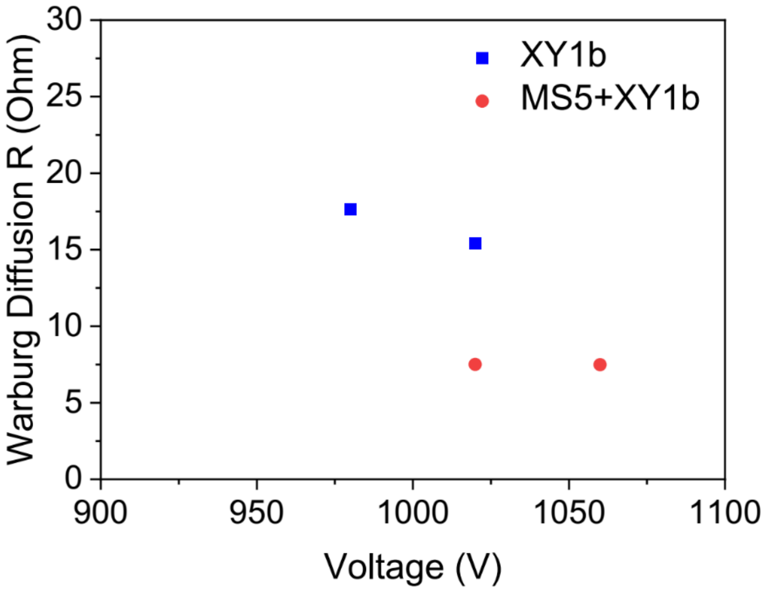

Supplementary Fig. 10 | Warburg diffusion resistance of the dye sensitized solar cells (DSCs) based on the dyes XY1b and MS5+XY1b.

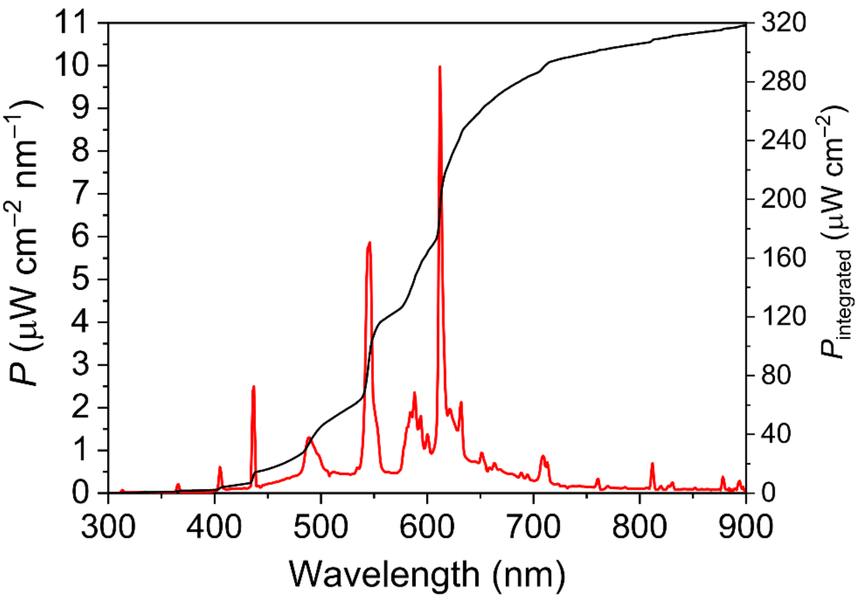

Supplementary Fig. 11 | Emitted power density spectrum (*P*, red curve) of the model Osram 930 Warm White fluorescent tube light (Light intensity: 1000 lux). The black curve is the integrated power density ($P_{\text{integrated}}$).

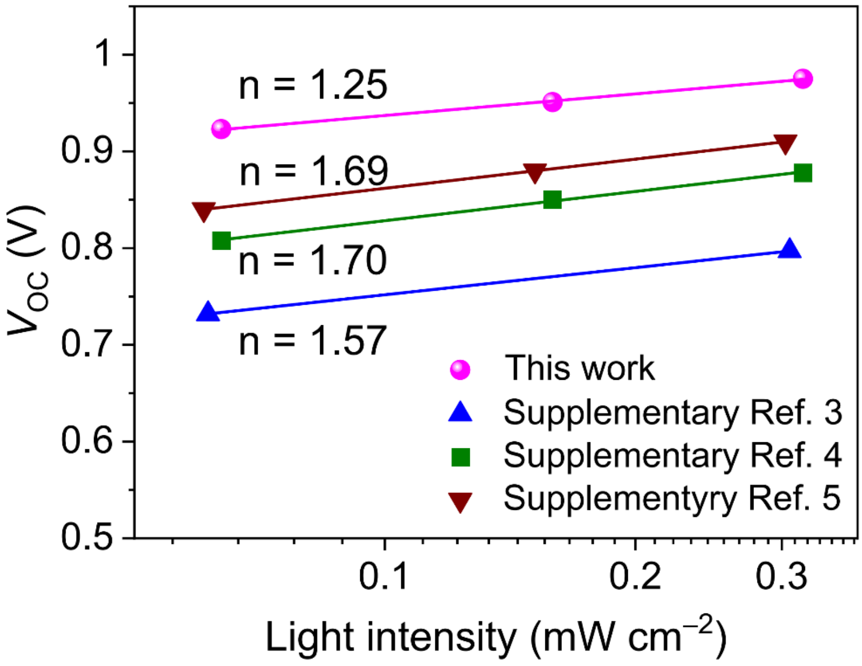

Supplementary Fig. 12 | Summarized the ideality factors (*n*) of representative works of dye sensitized solar cell (DSC) under ambient light. V_{oc} stands for the open circuit photovoltage.

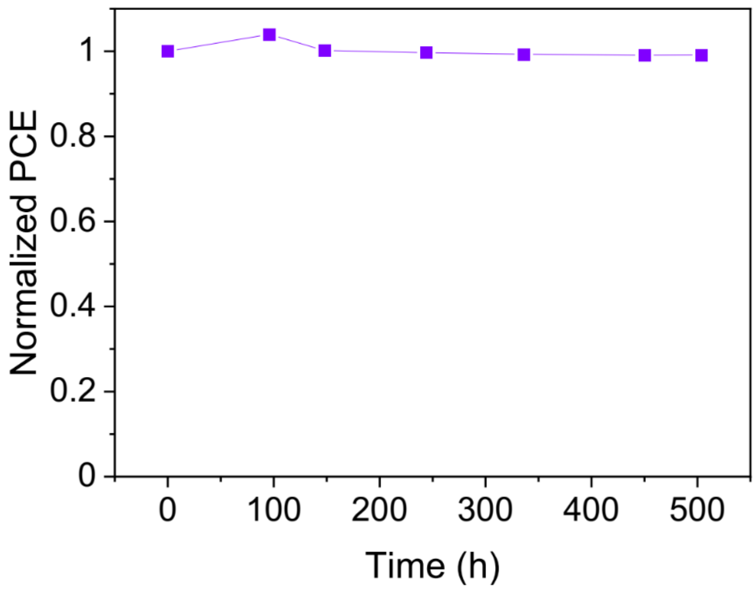

Supplementary Fig. 13 | The stability of the dye sensitized solar cell (DSC) based on dyes MS5+XY1b with a photoactive area of 2.80 cm^2 under continuous illumination by the indoor light (the model Osram 930 Warm White fluorescent tube light, light intensity: 1000 lux) at 25–30 °C. The device was encapsulated, and the power conversion efficiency (PCE) is normalized to its initial value of 34.5% at the start of the lifetime test.

Supplementary Table 1. Optical and electrochemical properties of the dyes NT35, MS4, MS5, and XY1b.

Dyes	ε ($\times 10^4$ M^{-1} cm^{-1}) ($\lambda_{abs,max}^{sol}$) $^{a)}$	$\lambda_{abs,max}^{film}$ $^{b)}$ (nm)	E_{0-0} $^{c)}$ (eV)	E_{ox} $^{d)}$ (V vs. Fc$^+$/Fc)	E_{ox} $^{e)}$ (V vs. SHE)	E_{red} $^{f)}$ (V vs. SHE)
NT35	3.28 (319 nm), 3.88 (434 nm)	422	2.48	0.581	1.21	−1.27
MS4	3.14 (330 nm), 0.79 (463 nm)	468	2.29	0.544	1.17	−1.12
MS5	4.50 (329 nm), 1.26 (463 nm)	468	2.28	0.555	1.18	−1.10
XY1b	3.40 (328 nm), 3.64 (542 nm)	531	1.85	0.459	1.08	−0.74

[a]Measured from dye dissolved in THF. [b]Measured from dyes adsorbed on the 2.2 µm-thick transparent TiO_2 films. [c]Calculated from the absorption onset (λ_{onset}) of the corresponding dye using the formula: $E_{0-0} = 1240/\lambda_{onset}$. [d]Measured from the cyclic voltammogram of the corresponding dye adsorbed on a 2.2 µm-thick transparent TiO_2 film in 0.1M LiTFSI/acetonitrile supporting electrolyte versus Ferrocene as external standard. More details in Supplementary Fig. 2. [e]The potential vs. ferrocene were converted to that vs. standard hydrogen electrode (SHE) by adding 0.624 V. [f]Calculated using: $E_{ox} - E_{0-0}$.

Supplementary Table 2. Photovoltaic parameters of dye sensitized solar cells (DSCs) based on copper redox shuttles under standard AM1.5G, 100 mW cm^{-2} condition.

Dyes	V_{oc} (mV)	J_{sc} (mA cm^{-2})	FF (%)	PCE (%)	Ref.
MS5/XY1b	1046	15.84	81.3	13.5	This work
XY1b/Y123	1050	15.74	79.0	13.1	3
HY64	1025	15.76	77.4	12.5	6
Y123	1090	13.65	78.0	11.6	3
XY1/L1	1080	15.9	67.0	11.5	5
XY1b	1010	14.98	76.0	11.5	3
XY1/D35	1030	16.19	68.0	11.3	4
L350	1140	13.00	76.0	11.2	7
Y123	1080	13.87	73.3	11.0	8
Y123	1040	15.53	64.0	10.3	9
XY1	1020	14.56	67.0	10.2	4
D35	1100	12.48	72.0	9.9	4
Y123	1070	13.17	66.0	9.3	8
LEG4	895	14.10	71.3	9.0	10
LEG4	1020	12.60	62.0	8.3	11
LEG4	1010	10.80	73.0	7.9	12
C218	932	11.29	66.0	7.0	13
LEG4	1035	9.40	67.0	6.0	14
L348	1170	6.40	72.0	5.3	7
G3	720	9.30	66.0	4.4	15

Dyes	V_{oc} (mV)	J_{sc} (mA cm^{-2})	FF (%)	PCE (%)	Ref.
D1	810	5.90	77.0	3.7	16
N719	790	3.20	55.0	1.4	17

Supplementary Table 3. Parameters of the current density-voltage (*J*–*V*) curves shown in Supplementary Fig. 6.

		V_{oc} (V)	J_{sc} (mA cm^{-2})	FF (%)	PCE (%)
XY1b	Measurement	1.014	15.26	76.3	11.8
	Quasi-SQ limit	1.370	17.26	90.8	21.5
	Transp. Limit	1.014	15.26	85.0	13.0
MS5+XY1b	Measurement	1.046	15.84	81.3	13.5
	Quasi-SQ limit	1.370	16.87	90.8	21.0
	Transp. Limit	1.046	15.84	86.4	14.3

Supplementary Table 4. Photovoltaic parameters of the DSC based on MS5+XY1b with photoactive area of 2.80 cm^2 using different redox couple ratios under 1,000 lux light intensity of an Osram 930 warm white fluorescent light.

Electrolyte components	P_{max} (µW)	PCE (%)	V_{oc} (mV)	I_{sc} (mA)	FF
0.20 M Cu(I) / 0.10 M Cu(II)[a]	281.3	31.6	888	0.392	0.808
0.20 M Cu(I) / 0.04 M Cu(II)[b]	301.1	33.8	957	0.388	0.813
0.10 M Cu(I) / 0.04 M Cu(II)[c]	307.4	34.5	975	0.387	0.815

[a]The copper-based electrolyte consisting of 0.2 M [Cu$^{(I)}$(tmby)$_2$]TFSI and 0.10 M [Cu$^{(II)}$(tmby)$_2$](TFSI)$_2$ complexes with 0.1 M LiTFSI and 0.6 M NMB in acetonitrile. [b]The copper-based electrolyte consisting of 0.2 M [Cu$^{(I)}$(tmby)$_2$]TFSI and 0.04 M [Cu$^{(II)}$(tmby)$_2$](TFSI)$_2$ complexes with 0.1 M LiTFSI and 0.6 M NMB in acetonitrile. [c]The copper-based electrolyte consisting of 0.1 M [Cu$^{(I)}$(tmby)$_2$]TFSI and 0.04 M [Cu$^{(II)}$(tmby)$_2$](TFSI)$_2$ complexes with 0.1 M LiTFSI and 0.6 M NMB in acetonitrile.

Supplementary Table 5. The best photovoltaic performance of various types of solar cell with the photoactive area of at least 1.0 cm^2 under ambient light conditions.

PV type	Area (cm^2)	Lighting source	Flux (lux)	P_{max} (μW cm^{-2})	V_{oc} (mV)	J_{sc} (μA cm^{-2})	FF (%)	PCE (%)	Ref.
DSC	2.80	Osram 930 Warm White Light	1000	109.8	975	138.2	81.5	34.5	This work
PSC	2.25	2700 K T2 fluorescent lamp	1000	84.27	/	/	/	30.6	18
OPV	1.0	2700 K LED lamp	1000	78.8	1100	90.6	79.1	26.1	19
GaAs	50[a]	OSRAM 827	1000	74.5	940	99.0	80.0	21.0	4
a-Si	3.18[b]	Compact fluorescent lamp	500	17.1	2.69	44.8	56.5	10.9	20

[a]Six pixels of each area of 8.33 cm^2 in parallel and in series connection

[b]Four cells in series

PSC: Metal halide perovskite

OPV: Organic polymers

GaAs: the solar cell was produced by Alta Devices

a-Si: the Sanyo amorphous silicon cell designed for indoors

Supplementary Table 6. Summarized open circuit photovoltages (*V_{oc}*) under different ambient light intensities and ideality factor (*n*) of benchmark works of DSCs.

Flux (lux)	V_{oc} (mV)	*n*	Ref.
1000<br>500<br>200	975<br>951<br>923	1.25	This work
1000<br>500<br>200	910<br>880<br>840	1.69	5
1000<br>500<br>200	878<br>850<br>808	1.70	3
100<br>500<br>200	797<br>/<br>732	1.57	4

Supplementary References

1. Vallot R., N'diaye A., Bermont A., Jakubowicz C., & Yu L.T. Comportement electrochimique en milieu aqueux de composes organiques moleculaires insolubles ou peu solubles—I. Voltamperometrie de derives quinoniques. *Electrochim. Acta* **25,** 1501−1512 (1980).
2. Pavlishchuk V. V. & Addison A. W. Conversion constants for redox potentials measured versus different reference electrodes in acetonitrile solutions at 25 °C. *Inorganica Chimica Acta* **298,** 97−102 (2000).
3. Cao, Y., Liu, Y., Zakeeruddin, S.M., Hagfeldt, A. & Grätzel, M. Direct contact of selective charge extraction layers enables high-efficiency molecular photovoltaics. *Joule* **2,** 1108–1117 (2018).
4. Freitag, M. et al. Dye-sensitized solar cells for efficient power generation under ambient lighting. *Nat. Photonics* **11,** 372–378 (2017).
5. Michaels, H. *et al.* Dye-sensitized solar cells under ambient light powering machine learning: towards autonomous smart sensors for the internet of things. *Chem. Sci.* **11,** 2895−2906 (2020).
6. Jiang, H. *et al.* Phenanthrene-fused-quinoxaline as a key building block for highly efficient and stable sensitizers in copper-electrolyte-based dye-sensitized solar cells. *Angew. Chem. Int. Ed.* **59,** 9324–9329 (2020).
7. Liu Y., et al. Electron-affinity-triggered variations on the optical and electrical properties of dye molecules enabling highly efficient dye-sensitized solar cells. *Angew. Chem. Int. Ed.* **57,** 14125−14128 (2018).
8. Cao, Y. *et al.* 11% efficiency solid-state dye-sensitized solar cells with copper(II/I) hole transport materials. *Nat. Commun.* **8,** 15390 (2017).
9. Saygili Y., et al. Copper bipyridyl redox mediators for dye-sensitized solar cells with high photovoltage. *J. Am. Chem. Soc.* **138,** 15087–15096 (2016).
10. Cong, J. et al. Bis(1,1-bis(2-pyridyl)ethane)copper(I/II) as an efficient redox couple for liquid dye-sensitized solar cells. *J. Mater. Chem. A* **4,** 14550−14554 (2016).
11. Freitag, M. et al. Copper phenanthroline as a fast and high-performance redox mediator for dye-sensitized solar cells. *J. Phys. Chem. C* **120,** 9595–9603 (2016).
12. García-Rodríguez, R., Jiang, R., Canto-Aguilar, E. J., Oskam, G. & Boschloo, G. Improving the mass transport of copper-complex redox mediators in dye-sensitized

solar cells by reducing the inter-electrode distance. *Phys. Chem. Chem. Phys.* **19,** 32132−32142 (2017).

13. Bai, Y. et al. High-efficiency organic dye-sensitized mesoscopic solar cells with a copper redox shuttle. *Chem. Commun.* **47,** 4376–4378 (2011).
14. Freitag, M. et al. High-efficiency dye-sensitized solar cells with molecular copper phenanthroline as solid hole conductor. *Energy Environ. Sci.* **8,** 2634−2637 (2015).
15. Magni, M. et al. Tetracoordinated bis-phenanthroline copper-complex couple as efficient redox mediators for dye solar cells. *Inorg. Chem.* **55,** 5245–5253 (2016).
16. Colombo, A. et al. Coupling of zinc porphyrin dyes and copper electrolytes: A springboard for novel sustainable dye-sensitized solar cells. *Inorg. Chem.* **56,** 14189–14179 (2017).
17. Hattori, S., Wada, Y., Yanagida, S. & Fukuzumi, S. Blue copper model complexes with distorted tetragonal geometry acting as effective electron-transfer mediators in dye-sensitized solar cells. *J. Am. Chem. Soc.* **127,** 9648–9654 (2005).
18. Cheng, R. et al. Tailoring triple-anion perovskite material for indoor light harvesting with restrained halide segregation and record high efficiency beyond 36%. *Adv. Energy Mater.* **9,** 1901980 (2019).
19. Cui, Y. *et al.* Wide-gap non-fullerene acceptor enabling high-performance organic photovoltaic cells for indoor applications. *Nat. Energy* **4,** 768−775 (2019).
20. De Rossi, F., Pontecorvo, T. & Brown, T. M. Characterization of photovoltaic devices for indoor light harvesting and customization of flexible dye solar cells to deliver superior efficiency under artificial lighting. *Appl. Energy* **156**, 413−422 (2015).

Supplementary Note 2

Synthesis of the dyes part

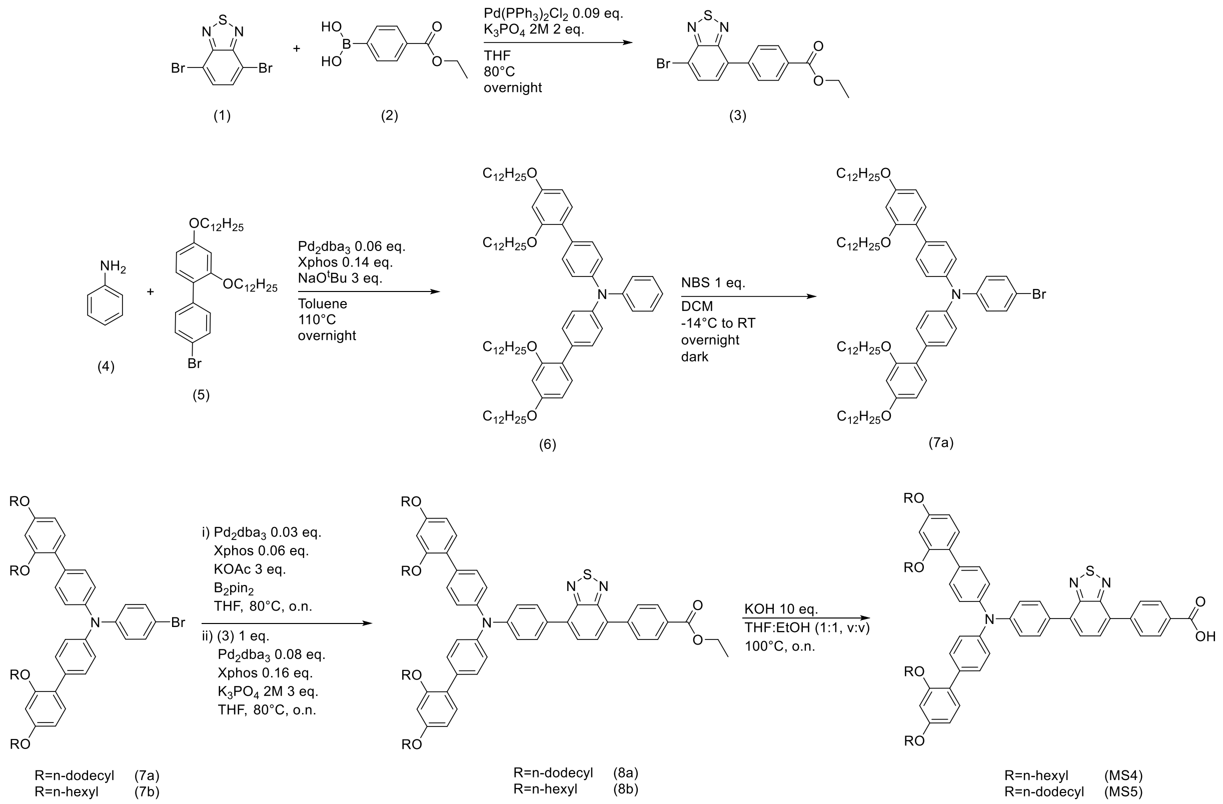

Supplementary Fig. 14 | Complete synthesis of MS4 and MS5.

Synthesis of Ethyl 4-(7-bromobenzo[*c*][1,2,5]thiadiazol-4-yl)benzoate (3)

4-ethoxycarbonylphenylboronic acid(1) (550 mg, 2.83 mmoles, 1 eq.), 4,7-dibromo-2,1,3-benzothiadiazole(2) (1 g, 3.4 mmoles, 1.2 eq.) and potassium phosphate tribasic (2M, 2.83 mL, 5.67 mmoles, 2 eq.) were charged in a schlenk tube with 5 mL anyhdrous THF and degassed three times. Then bis(triphenylphosphine)palladium(II) chloride (179 mg, 255 µmol, 0.09 eq.) was added and the reaction mixture was refluxed at 80°C overnight under Argon atmosphere. After cooling down to room temperature, the solution was diluted twice with diethyl ether and flashed through a pad of magnesium sulfate. The solvents were removed, and the crude purified by Silica gel flash chromatography eluting with hexanes to 30% hexanes in DCM. The desired product(3) was obtained as a yellow fluffy solid(0.132 g, 13%).^{1}H NMR (400 MHz, $CDCl_3$) δ 8.16 – 8.10 (m, 2H), 7.89 (dd, *J* = 9.6, 7.9 Hz, 3H), 7.56 (d, *J* = 7.5 Hz, 1H), 4.36 (q, *J* = 7.1 Hz, 2H), 1.36 (t, *J* = 7.1 Hz, 3H).^{13}C NMR (101 MHz, $CDCl_3$) δ 166.27, 153.90, 152.90, 140.83, 132.89, 132.19, 130.50, 129.90, 129.12, 128.73, 114.20, 77.34, 77.23, 77.02, 76.71, 61.15,

29.71, 14.37, 1.03.HRMS (APPI/LTQ-Orbitrap) m/z: $[M]^+$ Calcd for $C_{15}H_{11}BrN_2O_2S^+$ 361.9719; Found 361.9724.

Synthesis of *N*-(2',4'-bis(dodecyloxy)-[1,1'-biphenyl]-4-yl)-2',4'-bis(dodecyloxy)-*N*-phenyl-[1,1'-biphenyl]-4-amine (6)

Aniline(4) (121 µL, 1.33 mmoles, 1 eq.), 4'-bromo-2,4-bis(dodecyloxy)-1,1'-biphenyl (2 g, 3.32 mmoles, 2.5 eq.)(5) and sodium tert-butoxide (319 mg, 3.32 mmoles, 2.5 eq.) were charged in a schlenk tube along with 13 mL anhydrous Toluene and degassed three times. Then, tris(dibenzylideneacetone)dipalladium(0) (73 mg, 79.8 µmol, 0.06 eq.) and 2-(dicyclohexylphosphino)-2',4',6'-triisopropylbiphenyl (88.7 mg, 186 µmol, 0.14 eq.) were added before refluxing the mixture at 110°C overnight under Argon atmosphere. After cooling down to room temperature, the solution was diluted twice with diethyl ether and flashed through a pad of Celite. The solvents were removed and the residue purified with Silica gel chromatography eluting with hexanes to 50% dichloromethane in hexanes. The desired compound(6) was obtained as a beige solid(1.3 g, 86%).^{1}H NMR (400 MHz, Acetone) δ 7.38 – 7.31 (m, 4H), 7.21 – 7.09 (m, 5H), 7.03 – 6.86 (m, 8H), 6.52 – 6.41 (m, 4H), 3.88 (q, *J* = 6.3 Hz, 8H), 1.71 – 1.55 (m, 8H), 1.44 – 1.05 (m, 23H), 0.73 (dt, *J* = 9.8, 6.8 Hz, 12H).^{13}C NMR (101 MHz, Acetone) δ 205.39, 205.22, 205.21, 205.19, 205.17, 205.00, 159.81, 157.06, 147.96, 145.95, 133.27, 130.56, 130.18, 129.26, 123.97, 123.24, 122.67, 122.64, 105.80, 100.22, 68.07, 67.71, 31.76, 29.57, 29.55, 29.52, 29.49, 29.48, 29.46, 29.38, 29.32, 29.24, 29.22, 29.20, 29.18, 29.13, 29.10, 29.09, 28.99, 28.94, 28.88, 28.75, 28.55, 28.36, 26.05, 25.93, 22.46, 22.45, 13.51, 13.48.HRMS (nanochip-ESI/LTQ-Orbitrap) m/z: $[M]^+$ Calcd for $C_{78}H_{119}NO_4^+$ 1133.9134; Found 1133.9169. IR (ν_{max}, cm^{-1}) 2920 (s), 2851 (s), 1603 (m), 1494 (s), 1473 (s), 1304 (s), 1266 (s), 1173 (s), 1114 (m), 1022 (s), 1001 (m), 834 (s), 801 (s), 730 (m). Melting point: 51.4 – 53.2°C.

Synthesis of *N*-(2',4'-bis(dodecyloxy)-[1,1'-biphenyl]-4-yl)-*N*-(4-bromophenyl)-2',4'-bis(dodecyloxy)-[1,1'-biphenyl]-4-amine (7a)

N-(2',4'-bis(dodecyloxy)-[1,1'-biphenyl]-4-yl)-2',4'-bis(dodecyloxy)-*N*-phenyl-[1,1'-biphenyl]-4-amine(6) (1.26 g, 1.11 mmoles, 1 eq.) was dissolved in 15 mL dichloromethane, protected from light and cooled down to -14°C. Then, NBS (198 mg, 1.11 mmoles, 1 eq.) was added and the mixture allowed to warm up to room temperature overnight. The solvent was removed and hexanes was added to the crude to induce the formation of a white solid which was removed and discarded. Hexanes was removed and the desired product(7a) was obtained as a transparent wax and used for the next step without further purification.

Synthesis of Ethyl 4-(7-(4-(bis(2',4'-bis(hexyloxy)-[1,1'-biphenyl]-4-yl)amino)phenyl)benzo[*c*][1,2,5]thiadiazol-4-yl)benzoate (8b)

N-(2',4'-bis(hexyloxy)-[1,1'-biphenyl]-4-yl)-*N*-(4-bromophenyl)-2',4'-bis(hexyloxy)-[1,1'-biphenyl]-4-amine(7b) (450 mg, 513 µmol, 1 eq.), acetic acidpotassium salt (151 mg, 1.54 mmoles, 3 eq.) and bis(pinacolato)diboron (261 mg, 1.03 mmoles, 2 eq.) were charged in a schlenk tube with 5 mL anhydrous THF and degassed three times. Then tris(dibenzylideneacetone)dipalladium(0) (14.1 mg, 15.4 µmol, 0.03 eq.) and 2-(dicyclohexylphosphino)-2',4',6'-triisopropylbiphenyl (14.7 mg, 30.8 µmol, 0.06 eq.) were added and the reaction mixture refluxed at 80°C overnight under Argon atmosphere. After cooling down to room temperature, the solution was diluted twice with diethyl ether and flashed through a thin pad of Celite. The solvents were removed and 20 mL Ethanol was added to the residue to afford *N*-(2',4'-bis(hexyloxy)-[1,1'-biphenyl]-4-yl)-2',4'-bis(hexyloxy)-*N*-(4-(4,4,5,5-tetramethyl-1,3,2-dioxaborolan-2-yl)phenyl)-[1,1'-biphenyl]-4-amine as a beige wax, which was used for the next step without further purification. *N*-(2',4'-bis(hexyloxy)-[1,1'-biphenyl]-4-yl)-2',4'-bis(hexyloxy)-*N*-(4-(4,4,5,5-tetramethyl-1,3,2-dioxaborolan-2-yl)phenyl)-[1,1'-biphenyl]-4-amine (185 mg, 200 µmol, 1 eq.), ethyl 4-(7-bromobenzo[*c*][1,2,5]thiadiazol-4-yl)benzoate (72.7 mg, 200 µmol, 1 eq.) and potassium phosphate tribasic (2M, 413 µL, 826 µmol, 4.13 eq.) and 5 mL anyhdrous THF were charged in a schlenk tube and degassed three times. Then tris(dibenzylideneacetone)dipalladium(0) (14.7 mg, 16 µmol, 0.08 eq.) and 2-(dicyclohexylphosphino)-2',4',6'-triisopropylbiphenyl (15.3 mg, 32 µmol, 0.16 eq.) were added and the reaction mixture was refluxed at 80°C overnight under Argon atmosphere. After cooling down to room temperature, the solution was diluted twice with diethyl ether and flashed through a pad of magnesium sulfate. The solvents were removed and the residue was taken to the next step without further purification.

Synthesis of 4-(7-(4-(bis(2',4'-bis(hexyloxy)-[1,1'-biphenyl]-4-yl)amino)phenyl)benzo[*c*][1,2,5]thiadiazol-4-yl)benzoic acid (MS4)

Ethyl-4-(7-(4-(bis(2',4'-bis(hexyloxy)-[1,1'-biphenyl]-4-yl)amino)phenyl)benzo[*c*][1,2,5]thiadiazol-4-yl)benzoate(9b) (216 mg, 200 µmol, 1 eq.) and potassium hydroxide (112 mg, 2 mmoles, 10 eq.) were dissolved in 10 mL of a THF:Ethanol(1:1, v:v) mixture along with 5 drops of deionized water. The resulting reaction mixture was then refluxed at 100°C under Argon atmosphere overnight. After cooling down to room temperature, the reaction was quenched with 30 mL 1M hydrochloric acid and charged

into a separation funnel. The organics were extracted using dichloromethane (3x10 mL) before drying over magnesium sulfate and removing the solvent. The crude was purified using Silica gel flash chromatography eluting with dichloromethane and then 5% methanol in dichloromethane and 5 drops of acetic acid. The final product (MS4) was finally washed with methanol and obtained as a shiny red solid(94 mg, 45%).^{1}H NMR (600 MHz, $CDCl_3$) δ 8.31 (d, *J* = 8.3 Hz, 2H), 8.13 (d, *J* = 8.3 Hz, 2H), 7.97 – 7.91 (m, 2H), 7.88 (d, *J* = 7.4 Hz, 1H), 7.83 (d, *J* = 7.4 Hz, 1H), 7.53 – 7.48 (m, 4H), 7.37 – 7.32 (m, 2H), 7.31 (d, *J* = 9.0 Hz, 2H), 7.28 – 7.25 (m, 4H), 6.58 (dq, *J* = 5.6, 2.4 Hz, 4H), 4.01 (dt, *J* = 11.8, 6.6 Hz, 8H), 1.86 – 1.76 (m, 8H), 1.56 – 1.22 (m, 32H), 0.96 – 0.92 (m, 6H), 0.92 – 0.87 (m, 9H).^{13}C NMR (151 MHz, $CDCl_3$) δ 170.49, 159.71, 159.44, 157.69, 157.10, 155.89, 154.20, 154.08, 148.59, 148.07, 145.90, 145.59, 142.78, 135.86, 134.17, 133.71, 133.67, 132.22, 131.20, 131.02, 130.60, 130.44, 130.26, 130.16, 130.08, 129.35, 129.09, 128.99, 127.12, 126.33, 126.25, 124.48, 124.40, 123.03, 122.81, 122.73, 120.66, 120.44, 105.39, 104.67, 100.50, 100.26, 77.37, 77.16, 76.95, 68.96, 68.86, 68.52, 68.46, 68.24, 68.14, 37.77, 37.35, 34.54, 34.48, 34.28, 33.98, 32.07, 31.76, 31.69, 31.66, 31.60, 30.93, 30.44, 30.31, 30.19, 29.85, 29.80, 29.75, 29.60, 29.51, 29.45, 29.41, 29.32, 29.21, 27.37, 26.92, 26.87, 26.84, 26.78, 26.32, 26.30, 26.21, 26.11, 26.04, 25.96, 25.91, 25.83, 24.89, 24.22, 22.89, 22.84, 22.78, 22.73, 14.28, 14.21, 14.17, 1.17. HRMS (APPI/LTQ-Orbitrap) m/z: $[M + H]^+$ Calcd for $C_{67}H_{78}N_3O_6S^+$ 1052.5606; Found 1052.5658. IR (ν_{max}, cm^{-1}) 2924 (m), 2855 (m), 1688 (m), 1599 (s), 1492 (s), 1467 (m), 1266 (s), 1178 (s), 1135 (m), 1042 (m), 890 (m), 829 (s). Melting point: 128.7 – 130.5 °C.

Synthesis of Ethyl-4-(7-(4-(bis(2',4'-bis(dodecyloxy)-[1,1'-biphenyl]-4-yl)amino)phenyl)benzo[*c*][1,2,5]thiadiazol-4-yl)benzoate (8a)

N-(2',4'-bis(dodecyloxy)-[1,1'-biphenyl]-4-yl)-*N*-(4-bromophenyl)-2',4'-bis(dodecyloxy)-[1,1'-biphenyl]-4-amine(7a) (450 mg, 593 µmol, 1 eq.), acetic acidpotassium salt (233 mg, 2.37 mmoles, 4 eq.) and bis(pinacolato)diboron (452 mg, 1.78 mmoles, 3 eq.) were charged in a schlenk tube along with 5 mL anhydrous THF and degassed three times. Then, tris(dibenzylideneacetone)dipalladium(0) (27.2 mg, 29.7 µmol, 0.05 eq.) and 2-(dicyclohexylphosphino)-2',4',6'-triisopropylbiphenyl (31.1 mg, 65.3 µmol, 0.11 eq.) were added and the reaction mixture refluxed at 80°C overnight under Argon atmosphere. After cooling down to room temperature, the solution was diluted twice with diethyl ether and flashed through a thin pad of Celite. The solvents were removed and 20 mL Ethanol was added to the residue to afford *N*-(2',4'-bis(dodecyloxy)-[1,1'-biphenyl]-4-yl)-2',4'-bis(dodecyloxy)-*N*-(4-(4,4,5,5-tetramethyl-1,3,2-dioxaborolan-2-yl)phenyl)-[1,1'-biphenyl]-4-amine as a beige wax,

which was used for the next step without further purification. *N*-(2',4'-bis(dodecyloxy)-[1,1'-biphenyl]-4-yl)-2',4'-bis(dodecyloxy)-*N*-(4-(4,4,5,5-tetramethyl-1,3,2-dioxaborolan-2-yl)phenyl)-[1,1'-biphenyl]-4-amine (392 mg, 311 μmol, 1 eq.), ethyl 4-(7-bromobenzo[*c*][1,2,5]thiadiazol-4-yl)benzoate (113 mg, 311 μmol, 1 eq.), potassium phosphate tribasic (2M, 642 μL, 1.28 mmoles, 4.13 eq.) were charged in a schlenk tube along with 5 mL of anhydrous THF and degassed three times. Then, tris(dibenzylideneacetone)dipalladium(0) (22.8 mg, 24.9 μmol, 0.08 eq.) and 2-(dicyclohexylphosphino)-2',4',6'-triisopropylbiphenyl (23.7 mg, 49.8 μmol, 0.16 eq.) were added and the reaction mixture was refluxed at 80°C overnight under Argon atmosphere. After cooling down to room temperature, the solution was diluted twice with diethyl ether and flashed through a pad of magnesium sulfate. The solvents were removed, and the residue was taken to the next step without further purification.

Synthesis of 4-(7-(4-(bis(2',4'-bis(dodecyloxy)-[1,1'-biphenyl]-4-yl)amino)phenyl)benzo[*c*][1,2,5]thiadiazol-4-yl)benzoic acid (MS5)

Ethyl 4-(7-(4-(bis(2',4'-bis(dodecyloxy)-[1,1'-biphenyl]-4-yl)amino)phenyl)benzo[*c*][1,2,5]thiadiazol-4-yl)benzoate(9a) (440 mg, 310 μmol, 1 eq.) and potassium hydroxide (174 mg, 3.1 mmoles, 10 eq.) were dissolved in 10 mL of a THF:Ethanol(1:1, *v*:*v*) mixture along with 5 drops of deionized water. The resulting reaction mixture was then refluxed at 100°C under Argon atmosphere overnight. After cooling down to room temperature, the reaction was quenched with 30 mL 1M hydrochloric acid and charged into a separation funnel. The organics were extracted using dichloromethane (3x10 mL) before drying over magnesium sulfate and removing the solvent. The crude was purified using Silica gel flash chromoatography eluting with dichloromethane and then 5% methanol in dichloromethane and 5 drops of acetic acid. The final product (MS5) was finally washed with methanol and obtained as a shiny red solid(304 mg, 70%).^{1}H NMR (600 MHz, $CDCl_3$) δ 8.33 (d, *J* = 8.3 Hz, 2H), 8.14 (d, *J* = 8.3 Hz, 2H), 7.97 – 7.93 (m, 2H), 7.89 (d, *J* = 7.4 Hz, 1H), 7.83 (d, *J* = 7.3 Hz, 1H), 7.56 – 7.49 (m, 4H), 7.36 (d, *J* = 8.6 Hz, 2H), 7.33 – 7.29 (m, 2H), 7.28 (d, *J* = 8.3 Hz, 5H), 6.58 (d, *J* = 6.9 Hz, 4H), 4.01 (dt, *J* = 11.7, 6.5 Hz, 8H), 1.86 – 1.72 (m, 8H), 1.56 – 1.09 (m, 46H), 0.91 (t, *J* = 7.0 Hz, 6H), 0.87 (t, *J* = 7.0 Hz, 6H).^{13}C NMR (151 MHz, $CDCl_3$) δ 171.26, 159.60, 157.00, 156.98, 154.09, 153.97, 148.48, 148.10, 145.84, 145.48, 144.95, 142.76, 135.68, 134.07, 133.62, 133.56, 132.39, 131.05, 130.89, 130.71, 130.52, 130.32, 130.14, 129.97, 129.24, 128.97, 128.86, 126.99, 126.30, 126.22, 124.37, 122.91, 122.70, 120.39, 105.28, 100.41, 77.27, 77.05, 76.84, 68.42, 68.13, 37.55, 37.12, 34.21,

32.08, 32.05, 31.96, 31.94, 31.92, 31.90, 31.89, 31.88, 31.88, 31.87, 31.87, 31.85, 31.84, 31.83, 31.82, 31.82, 31.80, 31.80, 31.79, 31.78, 31.78, 31.77, 31.76, 31.75, 31.75, 31.74, 31.73, 31.73, 31.72, 31.71, 31.70, 31.69, 31.68, 31.47, 30.82, 29.87, 29.86, 29.84, 29.83, 29.82, 29.81, 29.80, 29.75, 29.74, 29.72, 29.69, 29.67, 29.66, 29.64, 29.62, 29.61, 29.60, 29.59, 29.58, 29.57, 29.57, 29.56, 29.55, 29.55, 29.54, 29.53, 29.53, 29.52, 29.51, 29.50, 29.49, 29.47, 29.47, 29.46, 29.45, 29.44, 29.43, 29.39, 29.36, 29.35, 29.33, 29.31, 29.29, 29.27, 29.26, 29.24, 29.15, 29.13, 29.08, 26.80, 26.75, 26.72, 26.66, 26.24, 26.22, 26.15, 26.13, 26.12, 26.04, 26.02, 25.94, 25.93, 25.90, 24.13, 22.85, 22.82, 22.76, 22.74, 22.71, 22.67, 22.62, 22.59, 14.41, 14.39, 14.38, 14.37, 14.37, 14.36, 14.35, 14.35, 14.34, 14.33, 14.33, 14.32, 14.31, 14.31, 14.30, 14.29, 14.28, 14.26, 14.25, 14.24, 14.18, 14.15, 14.10, 14.06, 14.04, 1.06.HRMS (APPI/LTQ-Orbitrap) m/z: $[M + H]^+$ Calcd for $C_{91}H_{126}N_3O_6S^+$ 1388.9362; Found 1388.9403. IR (ν_{max}, cm^{-1}) 2921 (s), 2851 (m), 1686 (s), 1599 (s), 1492 (s), 1467 (m), 1422 (m), 1282 (s), 1180 (s), 1132 (m), 1045 (m), 830 (s). Melting point: 90.5 – 92.6 °C.

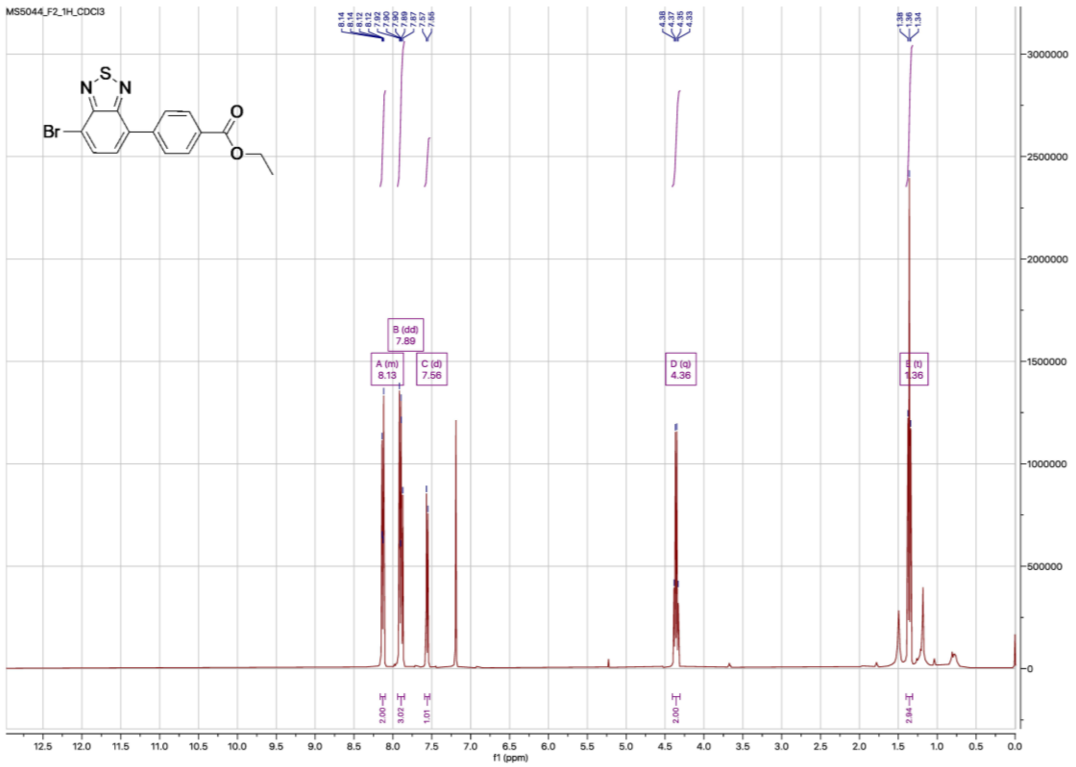

Supplementary Fig. 15 | ^{1}H spectrum of (3) in $CDCl_3$.

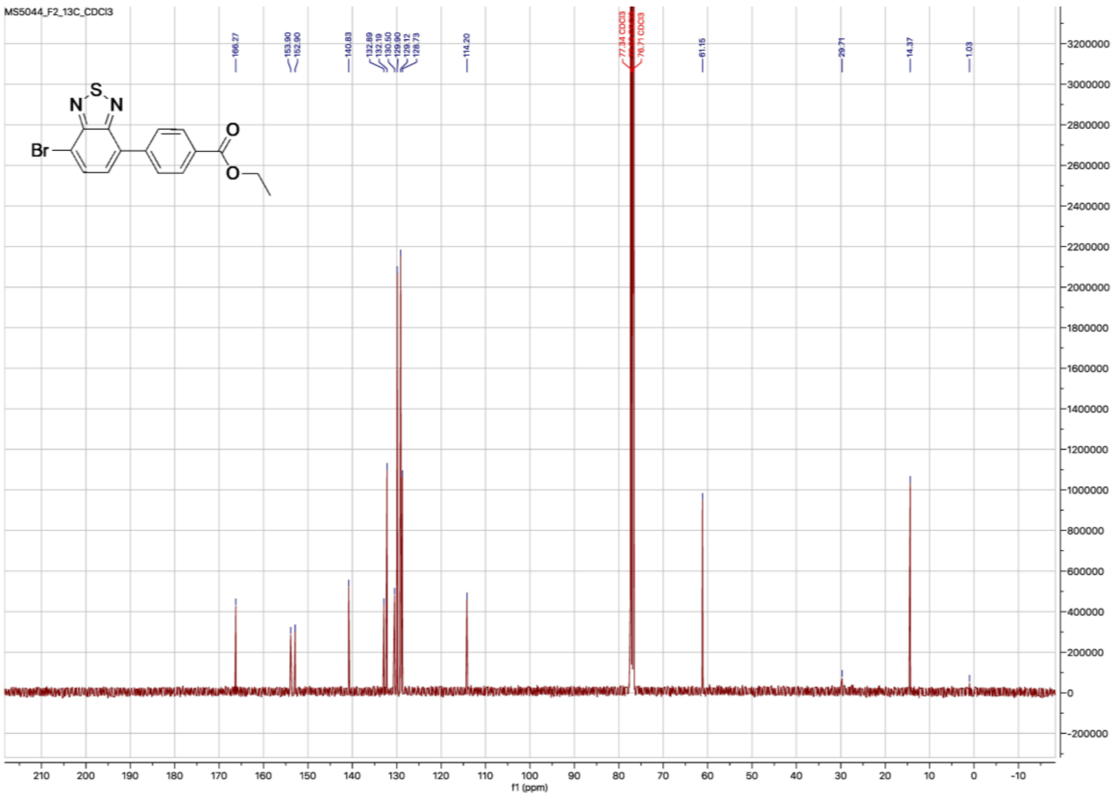

Supplementary Fig. 16 | ^{13}C spectrum of (3) in $CDCl_3$.

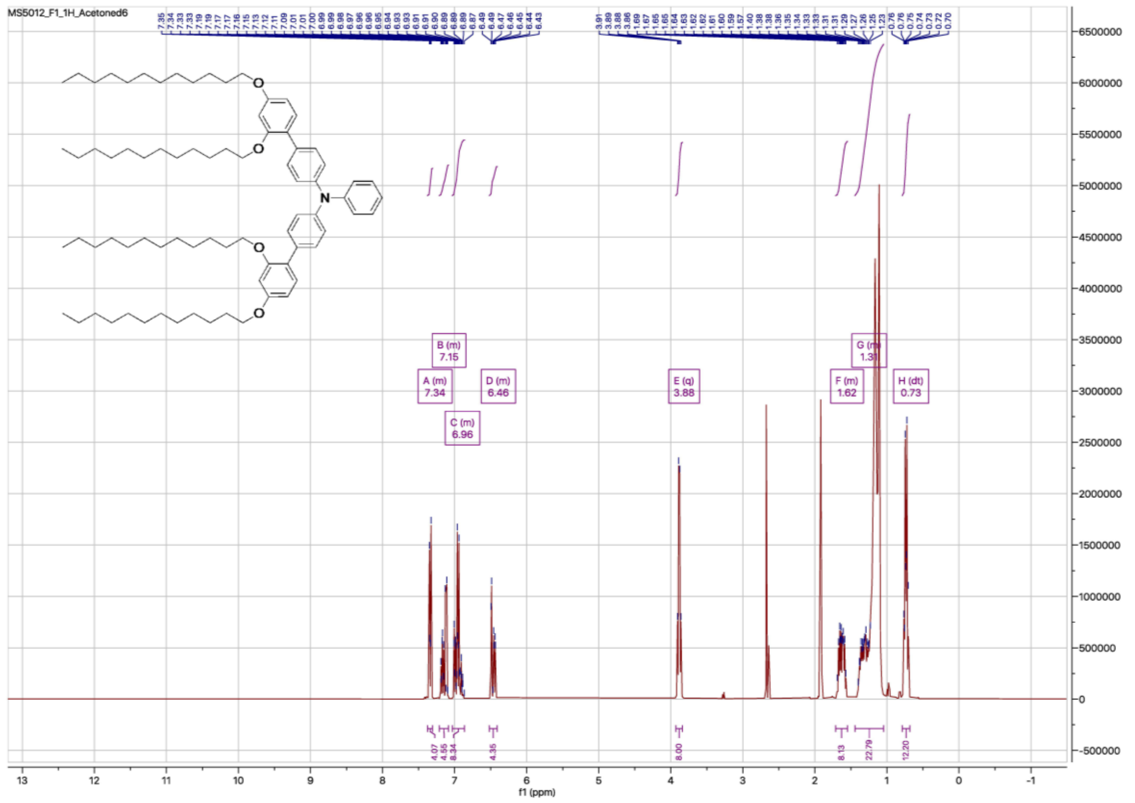

Supplementary Fig. 17 | ^{1}H spectrum of (10) in Acetone-d6.

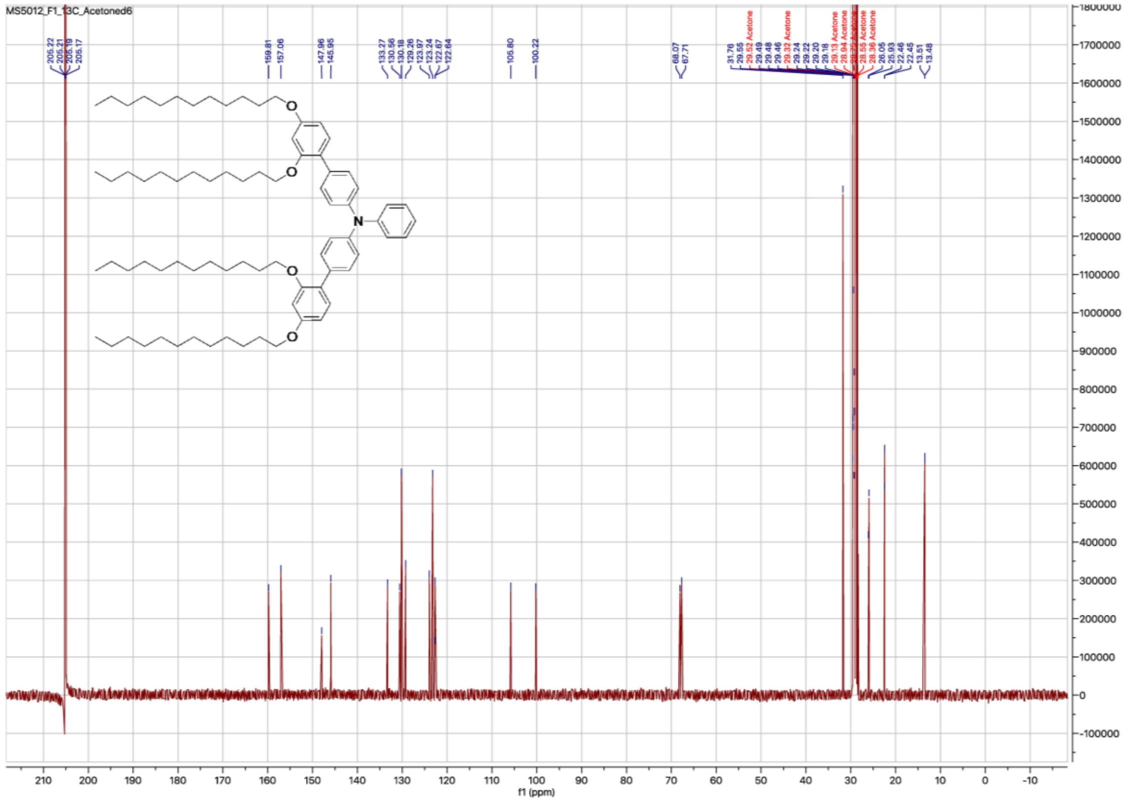

Supplementary Fig. 18 | ^{13}C spectrum of (10) in Acetone-d_6.

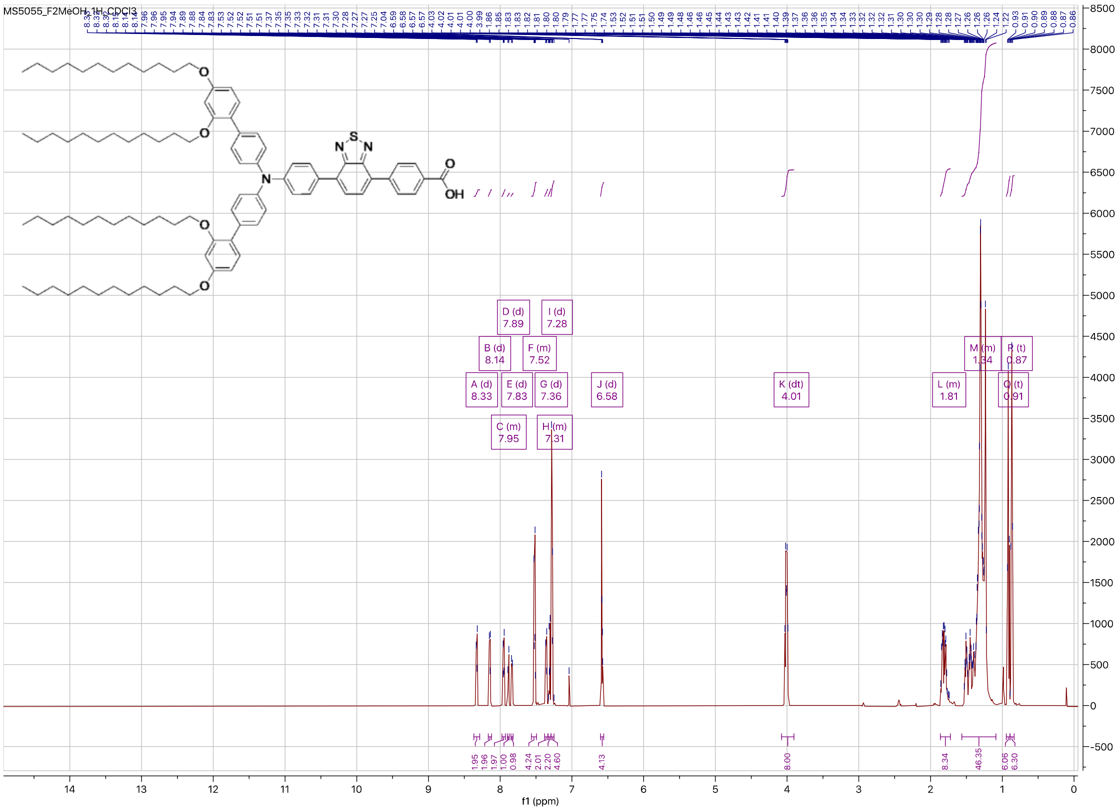

Supplementary Fig. 19 | ^{1}H spectrum of MS5 in $CDCl_3$.

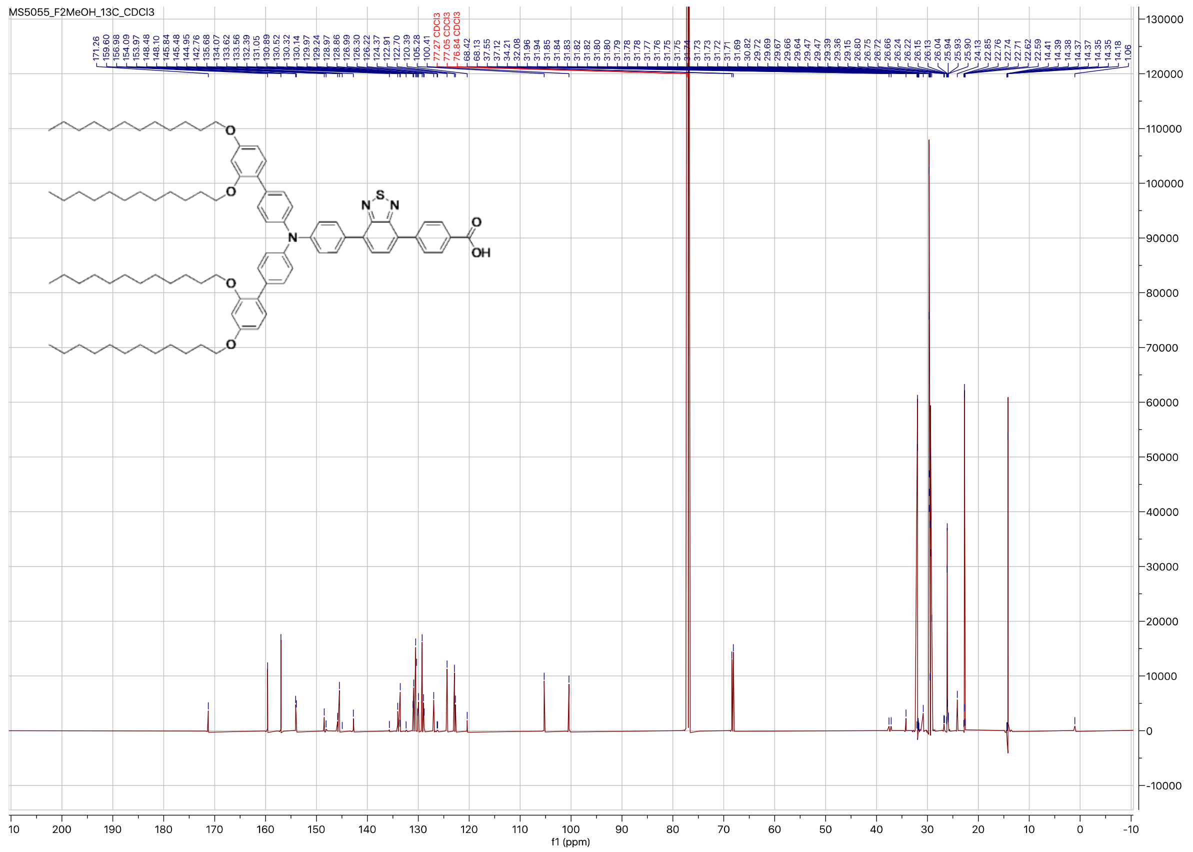

Supplementary Fig. 20 | ^{13}C spectrum of MS5 in $CDCl_3$.

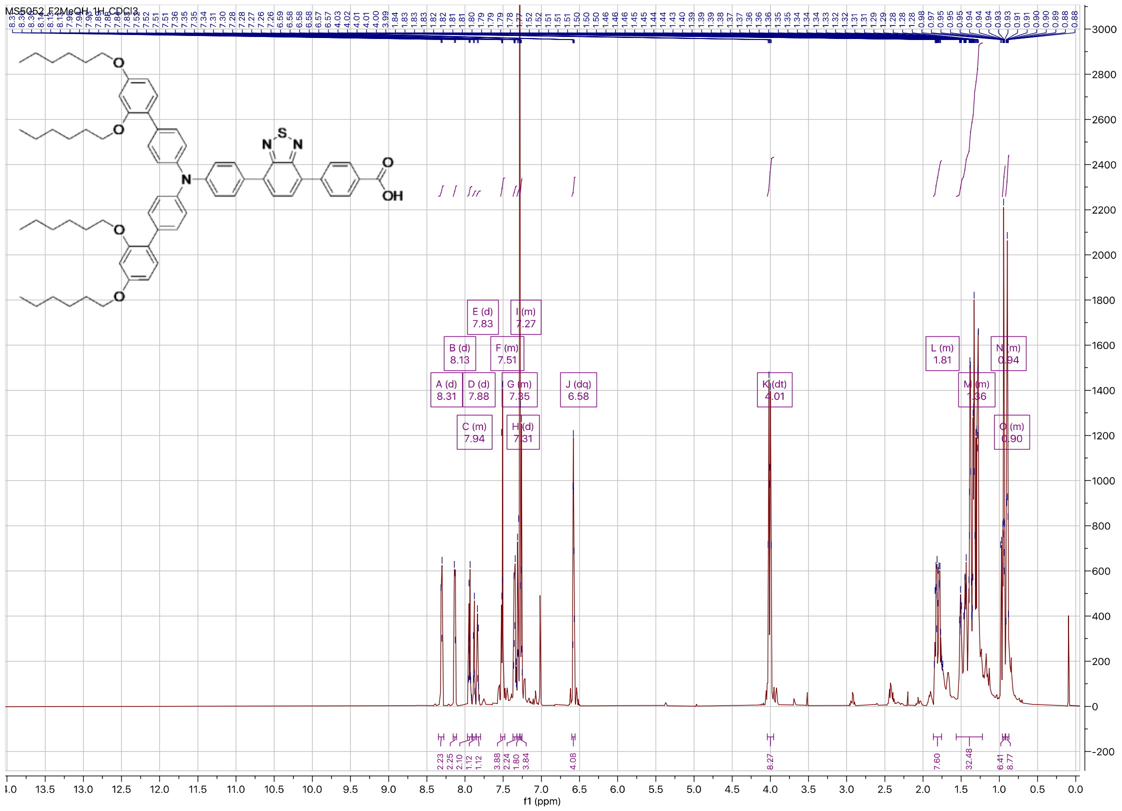

Supplementary Fig. 21 | ^{1}H spectrum of MS4 in $CDCl_3$.

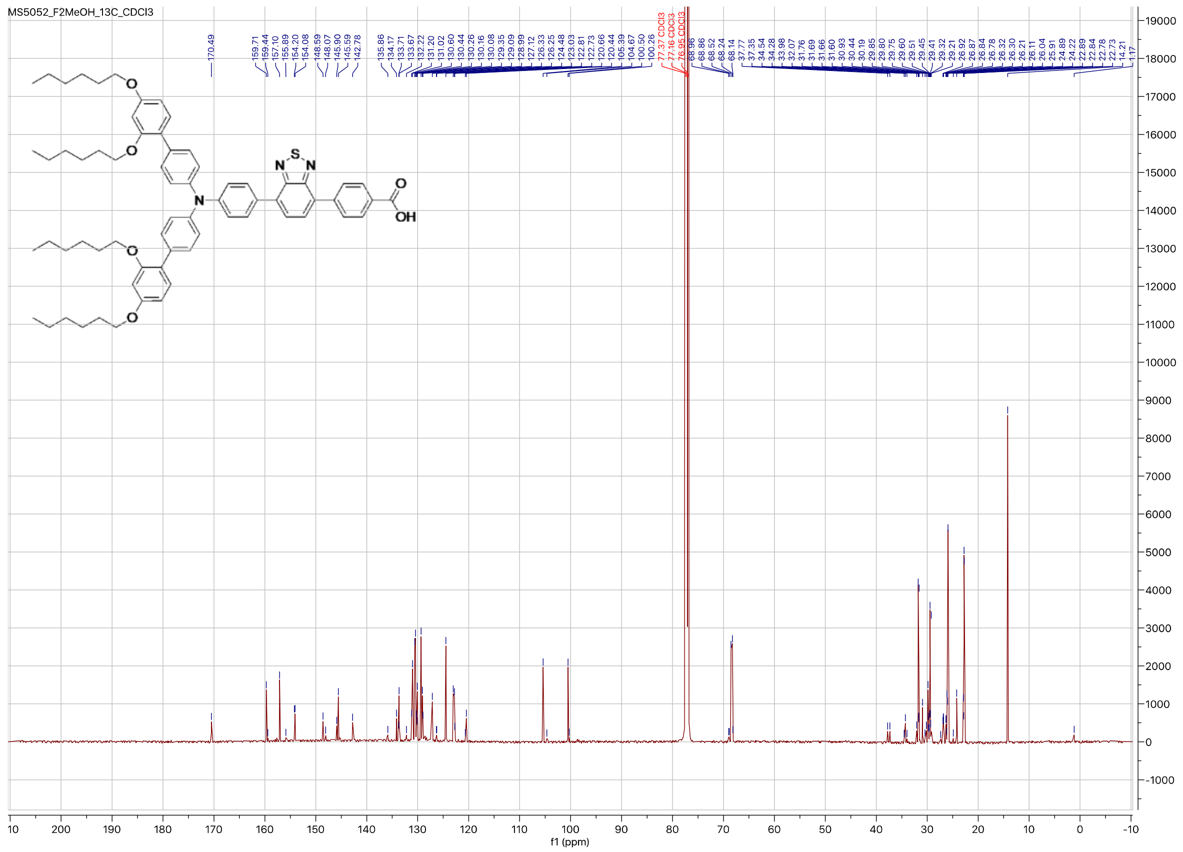

Supplementary Fig. 22 | ^{13}C spectrum of MS4 in CDCl3.
