# Supplementary material for: A molecular photosensitizer achieves a Voc of 1.24 V enabling highly efficient and stable dye-sensitized solar cells with copper(II/I)-based electrolyte
Source: Nat Commun. 2021 Mar 19;12:1777. doi: 10.1038/s41467-021-21945-3 (PMC7979847; doi:10.1038/s41467-021-21945-3)
Supplement: Supplementary file 3 — Solar Cells Reporting Summary [file 41467_2021_21945_MOESM3_ESM.pdf]

nature research

Corresponding author(s): Professor Michael Graetzel

Solar Cells Reporting Summary

Nature Research wishes to improve the reproducibility of the work that we publish. This form is intended for publication with all accepted papers reporting the characterization of photovoltaic devices and provides structure for consistency and transparency in reporting. Some list items might not apply to an individual manuscript, but all fields must be completed for clarity.

For further information on Nature Research policies, including our data availability policy, see Authors & Referees.

Experimental design

Please check: are the following details reported in the manuscript?

1. Dimensions

Item	Yes	No	Details
Area of the tested solar cells	☒	☐	The laboratory sized solar cell dimension: round, diameter 0.6 cm, area 0.28 cm2. Larger area solar cells: reactangular, 0.7cm x 4.0 cm, area 2.80 cm2, as described in the Experimental Section (Fabrication of solar cells) in the Supplementary Information.
Method used to determine the device area	☒	☐	The device areas are determined by the design of the screens for printing TiO2 films, as described in the Experimental Section (Fabrication of solar cells) in the Supplementary Information.

2. Current-voltage characterization

Item	Yes	No	Details
Current density-voltage (J-V) plots in both forward and backward direction	☒	☐	The J-V plots in both forward and backward direction were recorded, but not added to the publication, since we did not observe hysteresis or any other unusual behaviour during the characterization of the dye sensitized solar cells. Note that hysteresis is notorious for perovskite solar cells but is rarely seen in dye sensitized solar cells.
Voltage scan conditions<br>*For instance: scan direction, speed, dwell times*	☒	☐	Voltage scan conditions: reserve scan, 125 mV/s, 80 ms, as described in the Experimental Section (Characterization of Solar Cells) in the Supplementary Information.
Test environment<br>*For instance: characterization temperature, in air or in glove box*	☒	☐	Test environment: room temperature and humidity, in air, as described in the Experimental Section (Characterization of Solar Cells) in the Supplementary Information.
Protocol for preconditioning of the device before its characterization	☒	☐	Current-voltage characteristics were recorded in accordance with the procedure reported in the Experimental Section (Characterization of Solar Cells) in the Supplementary Information. No specific protocol for preconditioning was applied.
Stability of the J-V characteristic<br>*Verified with time evolution of the maximum power point or with the photocurrent at maximum power point; see ref. 7 for details.*	☐	☒	Our DSCs are stable under light soaking conditions.

3. Hysteresis or any other unusual behaviour

Item	Yes	No	Details
Description of the unusual behaviour observed during the characterization	☐	☒	We did not observe hysteresis or any other unusual behaviour during the characterization of the solar cells.
Related experimental data	☐	☒	Our DSCs have no hysteresis behavior.

4. Efficiency

Item	Yes	No	Details
External quantum efficiency (EQE) or incident photons to current efficiency (IPCE)	☒	☐	IPCE spectra were recorded, as reported in Fig. 2a and Fig. 3a of the manuscript.
A comparison between the integrated response under the standard reference spectrum and the response measure under the simulator	☒	☐	The integrated Jsc from IPCE spectra agrees well with the measured Jsc from JV measurements, as detailed in the manuscript (Fig. 2a, Fig. 3a, and Table 1).The mismatch between the Jsc calculated from the overlap integral of the IPCE with the standard AM1.5G emission spectrum (ASTM G173-03) and the one measured from standard AM1.5G solar simulator is very small.
For tandem solar cells, the bias illumination and bias voltage used for each subcell	☐	☒	We do not study tandem DSCs in the current work.

5. Calibration

Item	Yes/No	Details
Light source and reference cell or sensor used for the characterization	☒ Yes ☐ No	As described in the Experimental Section (Characterization of Solar Cells) in the Supplementary Information, for small area solar cells (0.28 cm2): the light source was a 300-W Xenon lamp (Oriel) equipped with a SchottK113 Tempax sunlight filter (Praezisions Glas & OptikGmbH) to match the emission spectrum of the lamp to the AM1.5G standard. Before each measurement, the exact light intensity was determined using a calibrated Si reference diode equipped with an infrared cut-off filter (KG-3, Schott); For large area solar cells (2.80 cm2): indoor light sources OSRAM Warm White 930 was used and the light intensities were calibrated by the light meter (TES-1334, TES).
Confirmation that the reference cell was calibrated and certified	☒ Yes ☐ No	The Si reference diode was certified and calibrated by Newport Corporation PV Lab, Bozeman, MT, USA.
Calculation of spectral mismatch between the reference cell and the devices under test	☒ Yes ☐ No	The spectral mismatch between our simulator and the AM 1.5 solar source was insignificant as the integrated current densities estimated from the IPCE spectra were in good agreement with the values obtained from the current density-voltage (J-V) curves as detailed in the manuscript. Spectra mismatch factor of 1 was used.

6. Mask/aperture

Item	Yes/No	Details
Size of the mask/aperture used during testing	☒ Yes ☐ No	For small solar cells (0.28 cm2), all measurements were conducted using a non-reflective metal mask with an aperture area of 0.158 cm2 to cover part of the active area of the device and avoid stray light capturing by our devices; For the larger area solar cells (2.80 cm2) under indoor illumination, the mask with rectangular areas of 3.80 cm2 was used, as detailed in the Experimental Section (Characterization of Solar Cells) in the Supplementary Information.
Variation of the measured short-circuit current density with the mask/aperture area	☐ Yes ☒ No	We haven't measured the cells with apertures of different sizes.

7. Performance certification

Item	Yes/No	Details
Identity of the independent certification laboratory that confirmed the photovoltaic performance	☐ Yes ☒ No	The results have not been certified yet by an independent accredited laboratory.
A copy of any certificate(s) *Provide in Supplementary Information*	☐ Yes ☒ No	Not applicable as we have not yet certified our devices by an independent accredited laboratory.

8. Statistics

Item	Yes/No	Details
Number of solar cells tested	☒ Yes ☐ No	At least 16 devices for each condition were tested.
Statistical analysis of the device performance	☒ Yes ☐ No	A statistical analysis of the performance is presented Fig. 3c.

9. Long-term stability analysis

Item	Yes/No	Details
Type of analysis, bias conditions and environmental conditions *For instance: illumination type, temperature, atmosphere humidity, encapsulation method, preconditioning temperature*	☒ Yes ☐ No	We stated the long-term stability in in the Experimental Section (Characterization of Solar Cells) in the Supplementary Information.
